# Methicillin Resistance Alters the Biofilm Phenotype and Attenuates Virulence in *Staphylococcus aureus* Device-Associated Infections

**DOI:** 10.1371/journal.ppat.1002626

**Published:** 2012-04-05

**Authors:** Clarissa Pozzi, Elaine M. Waters, Justine K. Rudkin, Carolyn R. Schaeffer, Amanda J. Lohan, Pin Tong, Brendan J. Loftus, Gerald B. Pier, Paul D. Fey, Ruth C. Massey, James P. O'Gara

**Affiliations:** 1 UCD School of Biomolecular and Biomedical Science, University College Dublin, Dublin, Ireland; 2 UCD Conway Institute of Biomolecular and Biomedical Research, University College Dublin, Dublin, Ireland; 3 Channing Laboratory, Department of Medicine, Brigham and Women's Hospital and Harvard Medical School, Boston, Massachusetts, United States of America; 4 Department of Biology and Biochemistry, University of Bath, Bath, United Kingdom; 5 Department of Pathology and Microbiology, University of Nebraska Medical Center, Omaha, Nebraska, United States of America; 6 Department of Internal Medicine, University of Nebraska Medical Center, Omaha, Nebraska, United States of America; University of California, San Francisco, United States of America

## Abstract

Clinical isolates of *Staphylococcus aureus* can express biofilm phenotypes promoted by the major cell wall autolysin and the fibronectin-binding proteins or the *icaADBC*-encoded polysaccharide intercellular adhesin/poly-*N*-acetylglucosamine (PIA/PNAG). Biofilm production in methicillin-susceptible *S. aureus* (MSSA) strains is typically dependent on PIA/PNAG whereas methicillin-resistant isolates express an Atl/FnBP-mediated biofilm phenotype suggesting a relationship between susceptibility to β-lactam antibiotics and biofilm. By introducing the methicillin resistance gene *mecA* into the PNAG-producing laboratory strain 8325-4 we generated a heterogeneously resistant (HeR) strain, from which a homogeneous, high-level resistant (HoR) derivative was isolated following exposure to oxacillin. The HoR phenotype was associated with a R_602_H substitution in the DHHA1 domain of GdpP, a recently identified c-di-AMP phosphodiesterase with roles in resistance/tolerance to β-lactam antibiotics and cell envelope stress. Transcription of *icaADBC* and PNAG production were impaired in the 8325-4 HoR derivative, which instead produced a proteinaceous biofilm that was significantly inhibited by antibodies against the *mecA*-encoded penicillin binding protein 2a (PBP2a). Conversely excision of the SCC*mec* element in the MRSA strain BH1CC resulted in oxacillin susceptibility and reduced biofilm production, both of which were complemented by *mecA* alone. Transcriptional activity of the accessory gene regulator locus was also repressed in the 8325-4 HoR strain, which in turn was accompanied by reduced protease production and significantly reduced virulence in a mouse model of device infection. Thus, homogeneous methicillin resistance has the potential to affect *agr*- and *icaADBC*-mediated phenotypes, including altered biofilm expression and virulence, which together are consistent with the adaptation of healthcare-associated MRSA strains to the antibiotic-rich hospital environment in which they are frequently responsible for device-related infections in immuno-compromised patients.

## Author Summary

The acquisition of *mecA*, which encodes penicillin binding protein 2a (PBP2a) and methicillin resistance, by *Staphylococcus aureus* has added to an already impressive array of virulence mechanisms including enzyme and toxin production, biofilm forming capacity and immune evasion. And yet clinical data does not indicate that healthcare-associated methicillin resistant *S. aureus* (MRSA) strains are more virulent than their methicillin-susceptible counterparts. Here our findings suggest that MRSA sacrifices virulence potential for antibiotic resistance and that expression of methicillin resistance alters the biofilm phenotype but does not interfere with the colonization of implanted medical devices *in vivo*. High level expression of PBP2a, which was associated with a mutation in the c-di-AMP phosphodiesterase gene *gdpP*, resulted in these pleiotrophic effects by blocking *icaADBC*-dependent polysaccharide type biofilm development and promoting an alternative PBP2a-mediated biofilm, repressing the accessory gene regulator and extracellular protease production, and attenuating virulence in a mouse device-infection model. Thus the adaptation of MRSA to the hospital environment has apparently focused on the acquisition of antibiotic resistance and retention of biofilm forming capacity, which are likely to be more advantageous than metabolically-expensive enzyme and toxin production in immunocompromised patients with implanted medical devices offering a route to infection.

## Introduction

Infections caused by healthcare-associated *Staphylococcus aureus* and methicillin resistant *S. aureus* (MRSA) pose a major threat to hospital patients. A significant risk factor for these healthcare-associated infections is the extensive use of implanted prosthetic biomaterials for diagnostic and therapeutic purposes, which can be colonized by staphylococci giving rise to device-related infections (DVIs) involving biofilms [1]. In addition to resistance to β-lactam antibiotics such as oxacillin, current chemotherapeutics for DVIs have limited effectiveness against biofilms.

The challenge of developing therapeutics to treat staphylococcal biofilm infections is compounded by the existence of multiple biofilm mechanisms in both *S. aureus* and *S. epidermidis*. Thus, although production of the exopolysaccharide polysaccharide intercellular adhesin (PIA) or polymeric *N*-acetyl-glucosamine (PNAG) synthesized and exported by proteins encoded by the *icaADBC* genes is common among clinical isolates of both species [2], [3], [4], [5], [6], *ica*-independent biofilm production has also been described under *in vitro* conditions [1]. Using clinical isolates of *S. aureus*, we reported that methicillin resistant *S. aureus* (MRSA) strains express an *icaADBC*-independent biofilm phenotype in vitro [3], [4], which is instead dependent on the fibronectin binding proteins (FnBPA and FnBPB) and the major autolysin (Atl) [6], [7]. Atl-dependent autolytic activity and extracellular DNA release are involved in the early stages of biofilm production by these MRSA isolates, whereas the FnBPs promote subsequent intercellular accumulation and biofilm maturation [6], [7]. Unlike MRSA, clinical isolates of methicillin susceptible *S. aureus* (MSSA) express a PNAG-dependent biofilm phenotype on hydrophilic surfaces and an Atl/PNAG-dependent biofilm on hydrophobic surfaces. Other staphylococcal surface proteins implicated in biofilm include the biofilm-associated protein (Bap, in bovine *S. aureus* isolates), accumulation-associated protein (Aap) of *S. epidermidis* and its *S. aureus* homologue SasG [8], [9], [10], [11], protein A [12], SasC [13] and the extracellular matrix binding protein (Embp) of *S. epidermidis* [14]. The growing number of bacterial factors involved in staphylococcal biofilm development underscores the importance of this phenotype to the pathogen and suggests that there may be redundancy between biofilm mechanisms in different clinical isolates or on different surfaces.

The methicillin resistance gene *mecA* encodes the low affinity penicillin binding protein 2a carried on a mobile staphylococcal cassette chromosomal *mec* element (SCC*mec*) [15] of which eight different types have been characterized to date [16]. Heterogeneity is a feature of *S. aureus* methicillin resistance [17], [18], [19]. Many *S. aureus* clinical isolates exhibit heterogeneous methicillin resistance (HeR) under laboratory growth conditions. In a HeR strain the majority of cells grown in the presence of a β-lactam antibiotic are susceptible to low concentrations of the drug, with only a subpopulation expressing higher-level resistance. However HeR strains become capable of expressing homogeneous resistance (HoR) after selection on elevated concentrations of β-lactam antibiotics or under specific growth conditions [20]. This transition from HeR to HoR is complex with mutations at the *fem* (factor essential for methicillin resistance), *aux* (auxiliary) and *tagO* loci all being implicated [21], [22], [23]. In addition, an oxacillin-induced increased SOS response was shown to increase the mutation rate during HeR to HoR selection in a mechanism dependent on the accessory gene regulator Agr [24], [25]. Nevertheless because HoR clinical isolates are not deficient in any of these accessory factors and because mutations at these loci alone are insufficient to explain HeR to HoR selection, the mechanism underpinning this phenomenon is clearly complex.

SCC*mec* elements can also carry resistance genes for other antibiotics and heavy metals as well as the *psm*-*mec* locus, which encodes a cytolysin termed phenol-soluble modulin-mec (PSM-mec) [26]. Carriage of the *psm-mec* locus from type II SCC*mec* elements attenuates virulence, suppresses colony spreading activity, reduces expression of the chromosomally encoded PSMα and promotes biofilm formation [26], [27], [28]. Furthermore both the *psm-mec* encoded RNA and the PSM-mec peptide contribute to the pleiotropic role of this locus [27], [28].

Our analysis of *S. aureus* clinical isolates identified a novel biofilm phenotype expressed by MRSA clinical isolates in which the major cell wall autolysin Atl and the fibronectin-binding proteins FnBPA and FnBPB have fundamental functions [3], [6], [7]. The Atl/FnBP biofilm phenotype appears to be absent or less prevalent among methicillin-susceptible *S. aureus* (MSSA) isolates, which produce PNAG-dependent biofilms in vitro. Interestingly the *psm-mec* locus from a type II SCC*mec* element increased expression of FnBPA in the MSSA strain Newman [28]. However because clinical MRSA isolates that produce an FnBP-dependent biofilm [4], [6] can contain either type II (*psm-mec* ^+^) or type IV (*psm-mec* ^−^) SCC*mec* elements [27], [28], it seems unlikely that carriage of the *psm-mec* locus alone can explain the expression of Atl/FnBP- or PNAG-dependent biofilm phenotypes by MRSA and MSSA clinical isolates, respectively. Furthermore when considered with earlier reports suggesting a correlation between β-lactam resistance and biofilm [29], [30], [31], [32] our data raise the question as to whether methicillin susceptibility itself influences biofilm and if so, how.

Here we investigated the impact of acquisition of PBP2a-induced homogeneous oxacillin resistance on biofilm and virulence in the laboratory MSSA strain 8325-4. Genetic changes associated with the HoR phenotype in 8325-4 were identified by whole genome sequencing. The biofilm phenotypes of 8325-4 and its HoR derivative were compared and the impact of HoR oxacillin resistance on transcription of the *icaADBC* and *agr* loci examined. The impact of loss of SCC*mec* and methicillin susceptibility on the biofilm phenotype of the MRSA strain BH1CC was also examined. Extracellular protease production by 8325-4 and 8325-4 HoR was measured and the virulence of both strains compared in a mouse model of device-related infection. Our data reveal that expression of homogeneous methicillin resistance in *S. aureus* influences the biofilm phenotype and attenuates virulence.

## Results

### Impact of induced oxacillin susceptibility on MRSA biofilm production

**Figure 1:**
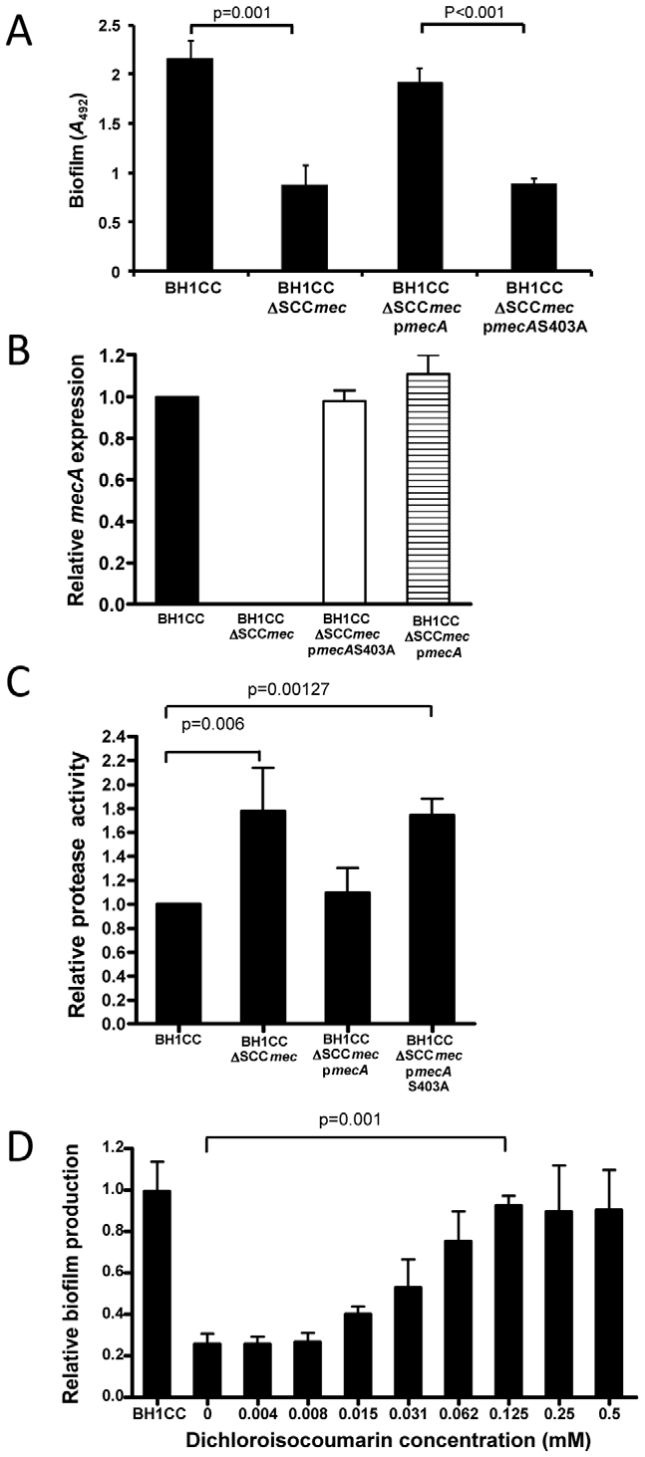
Impact of methicillin susceptibility on the MRSA biofilm phenotype. (A) Biofilm phenotypes of BH1CC, BH1CC ΔSCC*mec*, BH1CC ΔSCC*mec* p*mecA* and BH1CC ΔSCC*mec* p*mecA*S403A grown for 24 h at 37°C in BHI glucose in hydrophilic 96-well polystyrene plates. (B) Comparison of relative *mecA* transcription by real time RT-PCR in BH1CC, BH1CC ΔSCC*mec*, BH1CC ΔSCC*mec* p*mecA* and BH1CC ΔSCC*mec* p*mecA*S403A. Total RNA was extracted from cultures grown at 37°C to *A*_600_ = 1 (early exponential phase) in BHI glucose. Experiments were repeated at least three times and standard deviations are indicated. (C) Protease activity in culture supernatants of BH1CC, BH1CC ΔSCC*mec* and BH1CC ΔSCC*mec* p*mecA* grown for 8 h at 37°C in BHI glucose. (D) Relative biofilm production by BH1CC ΔSCC*mec* grown for 24 h at 37°C in BHI glucose media supplemented with the serine protease inhibitor dichloroisocoumarin (0–0.5 mM) in hydrophilic 96-well polystyrene plates compared to BH1CC (control). Experiments were repeated three times. Standard deviations and significance values are indicated.

To investigate the relationship between susceptibility to β-lactam antibiotics and the biofilm phenotype we used plasmid pSR_2_ carrying the *ccrAB* recombinase genes to promote excision of SCC*mec* in the MRSA clinical isolate BH1CC, which contains a type II SCC*mec* element and produces an Atl/FnBP-dependent biofilm in growth media supplemented with glucose [6], [7]. Excision of the SCC*mec* element in BH1CC resulted in a reduction in the oxacillin MIC from >100 µg/ml to <1 µg/ml (data not shown). Biofilm assays revealed a significant reduction in biofilm production by BH1CC ΔSCC*mec* in BHI glucose compared to BH1CC (Figure 1A). Complementation of the ΔSCC*mec* mutant with p*mecA* restored both oxacillin resistance (data not shown) and biofilm production in BHI glucose to near wild type levels (Figure 1A). In contrast p*mecA*S403A, in which the serine residue in the PBP2a active site was replaced with alanine (as described in the Materials and Methods), failed to restore oxacillin resistance (data not shown) or biofilm production in the BH1CC ΔSCC*mec* mutant (Figure 1A). RT-PCR analysis demonstrated that *mecA* mRNA levels were similar in the BH1CC SCC*mec* mutant carrying p*mecA* and p*mecA*S403A (Figure 1B) indicating that functional PBP2a and not *mecA* mRNA was responsible for the observed phenotypes.

Protease activity in the culture supernatant of BH1CC ΔSCC*mec* was reduced by approximately 50% compared to BH1CC and was restored to wild type levels by complementation with p*mecA* (Figure 1C). In addition the addition of the serine protease inhibitor dichloroisocoumarin to the growth media restored biofilm production by the BH1CC ΔSCC*mec* mutant strain in a dose-dependent manner (Figure 1D). Taken together these data indicate that oxacillin resistance promotes protein adhesin-dependent biofilm production in BH1CC at least in part by repressing extracellular protease production.

To determine if these findings could be extended to other clinical MRSA isolates, we deleted the SCC*mec* element from six clinical isolates in our collection that formed robust biofilm, were genetically amenable, had different SCC*mec* types and were from different clonal complexes. Furthermore we complemented all of the SCC*mec* mutants with the p*mecA* plasmid. Our data show that the impact of the SCC*mec* mutation on biofilm varied between the strains (Figure S1). In four strains biofilm production was significantly reduced whereas in the remaining two strains, biofilm was largely unaffected (Figure S1). However complementation with the p*mecA* plasmid increased biofilm production in all seven SCC*mec* mutants (Figure S1). The variable impact of the SCC*mec* deletions in different strains may reflect the complexity and multiple mechanisms of *S. aureus* biofilm production but nevertheless these data indicate that high level *mecA*/PBP2a expression always promoted biofilm production providing further evidence that methicillin resistance influences the biofilm phenotype in *S. aureus* clinical isolates.

### Isolation of a homogeneously oxacillin resistant derivative of *S. aureus* 8325-4

To investigate the impact of oxacillin resistance on *icaADBC*/PNAG-dependent *S. aureus* biofilm production, we generated a methicillin (oxacillin) resistant derivative of the laboratory strain 8325-4. 8325-4 was chosen because is it amenable to genetic manipulation and exclusively produces *icaADBC*/PNAG-dependent biofilms [4]. The related laboratory strain SH1000 (an *rsbU*-repaired derivative of 8325-4 [33]) is capable of PNAG-independent biofilm production [34], [35], while HG003 [36] (an *rsbU*- and *tcaR*-repaired derivative of 8325) exhibited a smooth colony morphology on Congo red agar and did not produce detectable levels of PNAG in our experiments (Figure S2).

**Figure 2:**
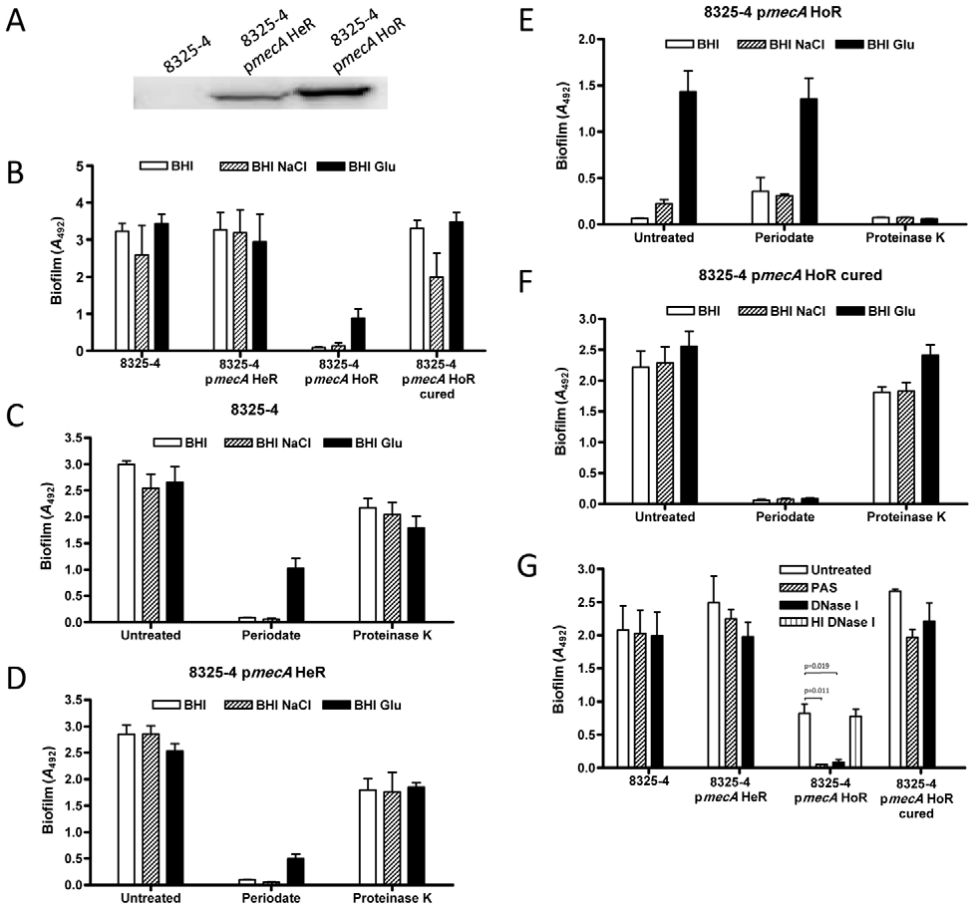
Impact of homogeneous oxacillin resistance on the biofilm phenotype of *S. aureus* 8325-4. (A) Western blot analysis of PBP2a levels in 8325-4, 8325-4 p*mecA* HeR and 8325-4 p*mecA* HoR. (B) Biofilm phenotypes of 8325-4, 8325-4 p*mecA* HeR, 8325-4 p*mecA* HoR and 8325-4 p*mecA* HoR (cured) grown for 24 h at 37°C in BHI, BHI NaCl and BHI glucose in hydrophilic 96-well polystyrene plates. (C–F) Dispersal of 8325-4 (C), 8325-4 p*mecA* HeR (D), 8325-4 p*mecA* HoR (E), 8325-4 p*mecA* HoR (cured) (F) biofilms grown for 24 h in BHI, BHI NaCl and BHI glucose by sodium metaperiodate and proteinase K. (G) Biofilm formation by 8325-4, 8325-4 p*mecA* HeR, 8325-4 p*mecA* HoR and 8325-4 p*mecA* HoR (cured) grown for 24 h in BHI glucose in the absence or presence of 500 µg/ml PAS, 0.25 mg/ml DNase I and heat-inactivated (HI) 0.25 mg/ml DNase I. Biofilm assays were repeated at least three times and standard deviations are indicated. * indicates a statistically significant difference p<0.01.

Transformation of 8325-4 with plasmid pRB474 carrying the *mecA* gene expressed from its own promoter (p*mecA*) was accompanied by heterogeneous resistance (HeR) to oxacillin (data not shown). A homogeneously, high-level oxacillin-resistant (HoR) derivative of 8325-4 p*mecA* was subsequently isolated on BHI media supplemented with 100 µg/ml oxacillin. Western blot analysis performed using commercial monoclonal antibody against PBP2a from Denka-Seiken (Japan) revealed substantially higher PBP2a levels in 8325-4 p*mecA* HoR than in 8325-4 p*mecA* HeR (Figure 2A). To investigate the genetic basis for the switch from expression of heterogeneous to homogeneous oxacillin resistance, the genomes of 8325-4 p*mecA* HeR and 8325-4 p*mecA* HoR were sequenced and aligned to the *S. aureus* NCTC8325 (CP000253) genome sequence as described in the Materials and Methods. Briefly one non-synonymous single nucleotide polymorphism (SNP) was identified in the *gdpP* (SAOUHSC_00015) gene of the HoR strain and confirmed by PCR amplification followed by capillary electrophoresis sequencing. This SNP results in a R_602_H substitution in GdpP, which has recently been identified as a c-di-AMP phosphodiesterase and implicated in resistance/tolerance to β-lactam antibiotics, biofilm formation and cell wall architecture [37], [38]. Subsequent characterization of nine independently isolated 8325-4 HoR strains identified a G_308_D substitution in seven strains, and Δ_382–504_ and Δ_80–174_ deletions in the other two strains. The GdpP R_602_ and G_308_ residues are highly conserved across multiple *S. aureus* species. These data revealed a strong correlation between high level PBP2a production and homogeneous oxacillin resistance in 8325-4 and suggest that *gdpP* mutations are also required for maximal PBP2a expression or stability in the 8325-4 p*mecA* HoR strain.

### Impact of *mecA*-induced oxacillin resistance on biofilm development

Comparison of the biofilm phenotypes of 8325-4, 8325-4 p*mecA* HeR and 8325-4 p*mecA* HoR under different growth conditions revealed that homogeneous oxacillin resistance was associated with a substantial reduction in biofilm forming capacity in BHI and BHI NaCl (*P*<0.0001) but that 8325-4 p*mecA* HoR retained the capacity to form reduced levels of biofilm in BHI glucose (Figure 2B). Our previous studies have shown that biofilm production by 8325-4 is dependent on PNAG under all growth conditions, whereas glucose-induced biofilm by MRSA isolates is mediated by protein adhesins [4], [6], [7]. Thus the loss of NaCl-induced biofilm was suggestive of impaired PNAG production in 8325-4 p*mecA* HoR. The reduced levels of biofilm produced by 8325-4 p*mecA* HoR compared to 8325-4 p*mecA* HeR in BHI glucose can be attributed to the altered biofilm phenotype expressed by this strain, but as described below, both strains formed similar levels of biofilm under flow conditions in BHI glucose. The biofilm phenotypes of 8325-4 and 8325-4 p*mecA* HeR were similar (Figure 2B) indicating the heterogeneous oxacillin resistance phenotype was not associated with a change in the biofilm phenotype. Curing the p*mecA* plasmid from 8325-4 p*mecA* HoR was associated with a return to oxacillin susceptibility and a restoration of the wild type biofilm phenotype (Figure 2B).

Sodium metaperiodate, which is known to break down PNAG-dependent biofilms, degraded 8325-4 and 8325-4 p*mecA* HeR biofilms grown in BHI, BHI NaCl and BHI glucose, whereas proteinase K had no significant effect (Figures 2C and D). In contrast 8325-4 p*mecA* HoR biofilms were completely dispersed by proteinase K (*P*<0.0001) but not by sodium metaperiodate (Figure 2E). Biofilms produced by 8325-4 p*mecA* HoR (cured) were dispersed by sodium metaperiodate but not proteinase K (Figure 2F). 8325-4 p*mecA* HoR biofilms were also inhibited by growth in the presence of DNase I whereas 8325-4 and 8325-4 p*mecA* HeR were unaffected (Figure 2G). Furthermore polyanethole sodium sulfonate (PAS), which blocks autolysis (and consequently eDNA release) [6], [7], completely blocked biofilm production by 8325-4 p*mecA* HoR but not 8325-4 and 8325-4 p*mecA* HeR (Figure 2G). Finally, independently isolated 8325-4 p*mecA* HoR isolates all exhibited the same biofilm phenotype (data not shown). Taken together these data indicate that acquisition of homogeneous oxacillin resistance is apparently accompanied by a switch from PNAG- to protein-mediated biofilm formation and that in contrast to 8325-4, the 8325-4 p*mecA* HoR biofilm phenotype is dependent on autolytic activity and eDNA release similar to that observed among clinical MRSA isolates expressing an Atl/FnBP-dependent biofilm [6], [7].

A BioFlux system was used to compare biofilm production by 8325-4 p*mecA* HeR and 8325-4 p*mecA* HoR grown in BHI NaCl and BHI glucose under flow conditions. These data showed that both strains formed abundant and similar levels of biofilm in BHI glucose (Figure S3). However neither strain was capable of biofilm production in BHI NaCl media (Figure S3) making it impossible to determine the contribution of PNAG to the biofilm phenotypes of both strains using the BioFlux system. The biofilm negative phenotype in BHI NaCl is likely due to the more hydrophobic surface of the BioFlux flow cell compared to the very hydrophilic, tissue-culture treated polystyrene used in our microtitre plate assay. We have previously reported that 8325-4 is incapable of producing a PNAG-type biofilm on hydrophobic polystyrene when grown in BHI NaCl, but can produce a complex biofilm dependent not only on PNAG but also protein adhesin(s) and eDNA on hydrophobic polystyrene in BHI glucose media [7].

### Contribution of the *icaADBC*, *fnbAB*, *atl* and *srtA* loci to 8325-4 p*mecA* HoR biofilm phenotype

**Figure 3:**
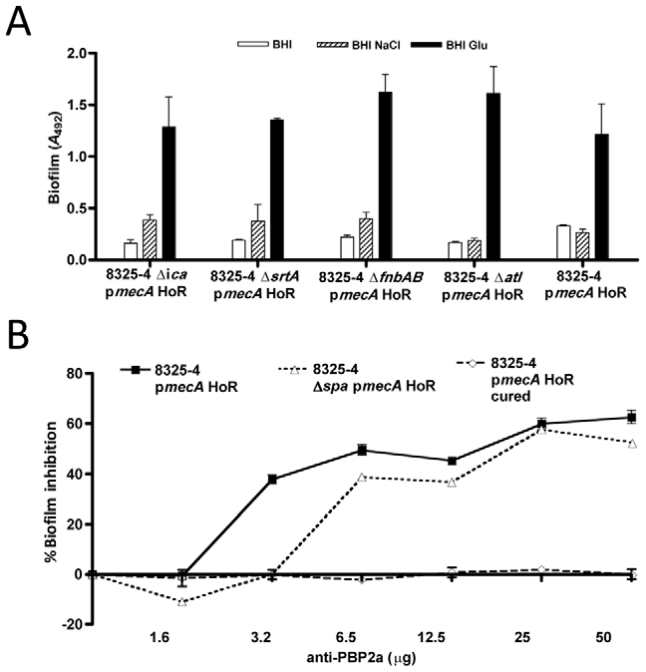
Contribution of PBP2a to the *S. aureus* biofilm phenotype. (A) Biofilm phenotypes of 8325-4 Δ*icaADBC*::Tc^r^ p*mecA* HoR, 8325-4 Δ*srtA*::Em^r^ p*mecA* HoR, 8325-4 Δ*fnbAB*::Tc^r^ p*mecA* HoR, 8325-4 Δ*atl*::Cm^r^ p*mecA* HoR and 8325-4 p*mecA* HoR (control) grown for 24 h at 37°C in BHI, BHI NaCl and BHI glucose in hydrophilic 96-well polystyrene plates. (B) Biofilm production by 8325-4 p*mecA* HoR, 8325-4 p*mecA* HoR (cured) and 8325-4 Δ*spa* p*mecA* HoR (control) grown in the presence of monoclonal anti-PBP2a antibody. Biofilms were grown at 37°C for 24 h in BHI glucose in hydrophilic 96-well polystyrene plates using monoclonal anti-PBP2a antibody. Experiments were repeated three times and average data are shown.

To investigate the genetic basis for protein-mediated biofilm in 8325-4 p*mecA* HoR, the p*mecA* plasmid was transformed into *fnbAB*::Tc^r^, *atl*::Cm^r^ and *srtA*::Tc^r^ derivatives of 8325-4 and homogeneous oxacillin resistant variants were isolated as described above. Biofilm assays revealed that HoR derivatives of 8325-4 *icaADBC*::Tc^r^, 8325-4 *fnbAB*::Tc^r^, 8325-4 *atl*::Cm^r^ and 8325-4 *srtA*::Tc^r^ exhibited a similar biofilm phenotype to 8325-4 p*mecA* HoR (Figure 3A). These data strongly suggest that the biofilm phenotypic switch associated with acquisition of oxacillin resistance is not dependent on PNAG, LPXTG cell wall anchored proteins (including FnBPA, FnBPB and Protein A) or the major autolysin.

This raised the possibility that overexpression of the PBP2a protein itself may promote biofilm development in BHI glucose. Consistent with this, commercial monoclonal antibodies (Calbiochem) to PBP2a reduced biofilm production by 8325-4 p*mecA* HoR by up to 50% (P<0.05)(Figure 3B). Biofilm production by 8325-4 Δ*spa* p*mecA* HoR was also inhibited by the PBP2a antibody in a concentration dependent manner (Figure 3B), ruling out any non-specific interference due to antibody binding to Protein A. In contrast the PBP2a monoclonal antibody had no significant effect on PNAG-dependent biofilm production by the plasmid-cured 8325-4 p*mecA* HoR strain (Figure 3B). These data directly implicate the PBP2a protein in 8325-4 p*mecA* HoR biofilm development. However it is important to note that the failure of the p*mecA*S403A plasmid to complement biofilm production in the BH1CC SCC*mec* strain (Figure 1A) also suggests that PBP2a-induced oxacillin resistance is required for the PBP2a-mediated biofilm phenotype

### Repression of PNAG production in 8325-4 p*mecA* HoR

**Figure 4:**
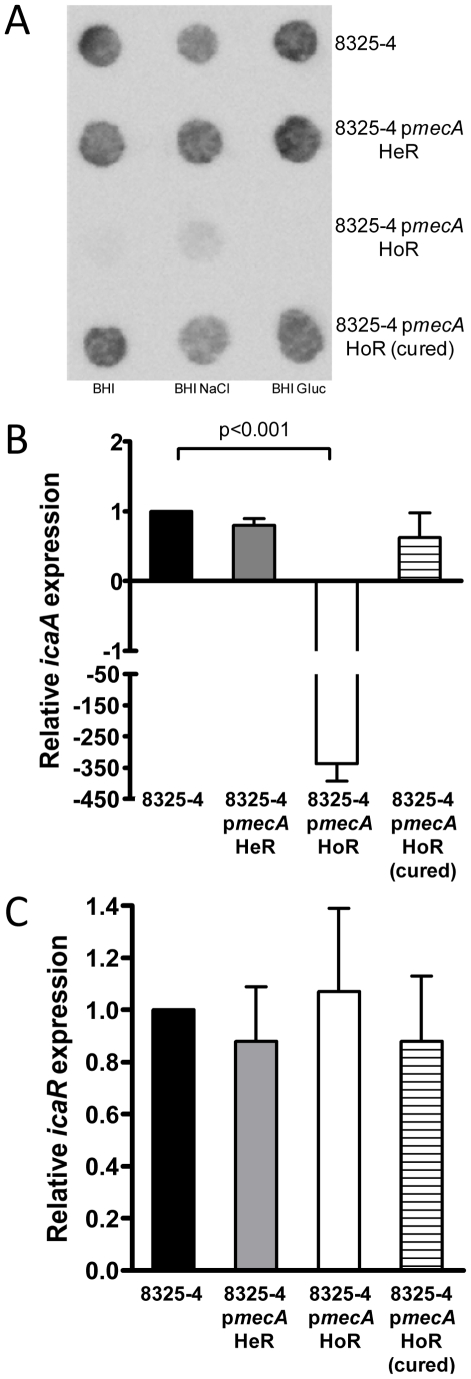
Impact of homogeneous oxacillin resistance on PNAG production in 8325-4. (A) Immunoblot analysis of PNAG production in whole cell extracts of 8325-4, 8325-4 p*mecA* HeR, 8325-4 p*mecA* HoR and 8325-4 p*mecA* HoR (cured) grown overnight at 37°C in BHI, BHI NaCl and BHI glucose. (B and C) Comparison of relative *icaA* (B) and *icaR* (C) transcription by real time RT-PCR in 8325-4, 8325-4 p*mecA* HeR, 8325-4 p*mecA* HoR and 8325-4 p*mecA* HoR (cured). Total RNA was extracted from cultures grown at 37°C to *A*_600_ = 1 (early exponential phase) in BHI glucose. Experiments were repeated at least three times and standard deviations are indicated. Statistical significance (p value) is indicated.

Comparison of 8325-4, 8325-4 p*mecA* HeR and 8325-4 p*mecA* HoR on Congo red agar revealed that the acquisition of homogeneous resistance was associated with a switch from a crusty to a smooth colony morphology, which is indicative of reduced PNAG production (data not shown). Accordingly immunoassays demonstrated that, unlike 8325-4 and 8325-4 p*mecA* HeR, PNAG was not produced by 8325-4 p*mecA* HoR (Figure 4A). Interestingly PNAG production was restored in the cured 8325-4 HoR strain (Figure 4A). Using real time RT-PCR, a >300-fold repression of *icaADBC* transcription was measured in 8325-4 p*mecA* HoR compared to 8325-4 (Figure 4B). Furthermore wild type levels of *icaADBC* transcription were restored in the plasmid-cured 8325-4 p*mecA* HoR strain (Figure 4B). Transcriptional activity of *icaR* was similar in both strains (Figure 4C). In addition genome sequencing of 8325-4 p*mecA* HoR confirmed the absence of any mutations in any known *ica* operon transcriptional regulator. Thus homogeneous oxacillin resistance alters the biofilm phenotype by repressing *icaADBC* transcription and PNAG production.

### Repression of extracellular protease production and Agr activity in 8325-4 p*mecA* HoR

**Figure 5:**
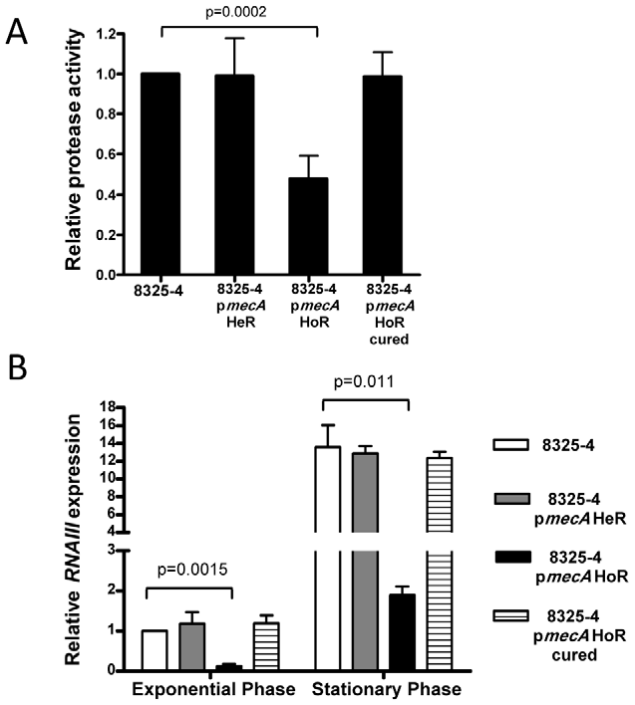
Impact of homogeneous oxacillin resistance on protease activity and expression of the accessory gene regulator locus in 8325-4. (A) Protease activity in culture supernatants of 8325-4, 8325-4 p*mecA* HeR, 8325-4 p*mecA* HoR and 8325-4 p*mecA* HoR (cured) grown for 8 h at 37°C in BHI glucose. (B) Comparison of relative *RNAIII* transcription by real time RT-PCR in 8325-4, 8325-4 p*mecA* HeR, 8325-4 p*mecA* HoR and 8325-4 p*mecA* HoR (cured). Total RNA was extracted from cultures grown at 37°C to *A*_600_ = 1 (early exponential phase) and for 8 hours (late exponential/early stationary phase; *A*_600_≈8) in BHI glucose. Experiments were repeated at least three times and standard deviations are indicated. Statistical significance (p value) is indicated.

Assays of protease activity in culture supernatants revealed an approximately 2-fold reduction in protease levels in 8325-4 p*mecA* HoR compared to 8325-4, 8325-4 p*mecA* HeR and the cured 8325-4 HoR strain (Figure 5A). Because extracellular protease activity is subject to regulation by the accessory gene regulator (Agr) system, we used real time RT-PCR to compare RNAIII transcript levels in these strains. These data revealed that RNAIII expression was significantly repressed in 8325-4 p*mecA* HoR grown to both the exponential and stationary phases of growth (Figure 5B). Genome sequence analysis confirmed the absence of any mutations in the *agr* locus of 8325-4 p*mecA* HoR. Thus the homogeneous oxacillin resistance phenotype in 8325-4 is associated with repression of the Agr system and extracellular protease production, both of which are consistent with PBP2a-mediated biofilm development by 8325-4 p*mecA* HoR. Furthermore these data correlate with our earlier observation that protease activity was increased in BH1CC ΔSCC*mec* culture supernatants (Figure 1B).

### Homogeneous methicillin resistance attenuates virulence in 8325-4

**Figure 6:**
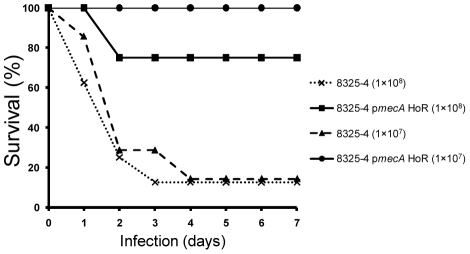
Impact of homogeneous oxacillin resistance on survival in a mouse device-related *S. aureus* infection model. 1-cm segments of 14-gauge polyethylene intravenous catheter were implanted into male 6-week old C57BL/6 mice (2 per mouse) and injected with 1×10^7^ or 1×10^8^ 8325-4 and 8325-4 p*mecA* HoR. Survival of the animals over seven days is plotted as a percentage of total numbers (*n* = 8 mice per group except for 8325-4 (1×10^7^) where n = 7 mice).

Because 8325-4 and 8325-4 p*mecA* HoR express PNAG-dependent and PNAG-independent biofilm phenotypes, respectively, their virulence was compared using an established mouse model of device-related infection [39], [40]. Briefly two 1-cm segments of 14-gauge polyethylene intravenous catheter were implanted subcutaneously per mouse. In total four groups of eight mice were inoculated with bacterial cell suspensions of 1×10^7^ or 1×10^8^ of either 8325-4 or 8325-4 p*mecA* HoR, injected directly into the catheter. These experiments revealed a significantly higher mortality rate among mice inoculated with 8325-4 compared to 8325-4 p*mecA* HoR (Figure 6). Seven of the eight mice inoculated with 1×10^8^ 8325-4 died or were euthanized by Day 4. Similarly only one of seven mice (one mouse failed to recover from the anesthetic) inoculated with 1×10^7^ 8325-4 survived beyond Day 4 (Figure 6). In contrast all eight animals inoculated with 1×10^7^ 8325-4 p*mecA* HoR survived to Day 7 and six of the eight animals inoculated with 1×10^8^ 8325-4 p*mecA* HoR survived to Day 7 (Figure 6).

**Figure 7:**
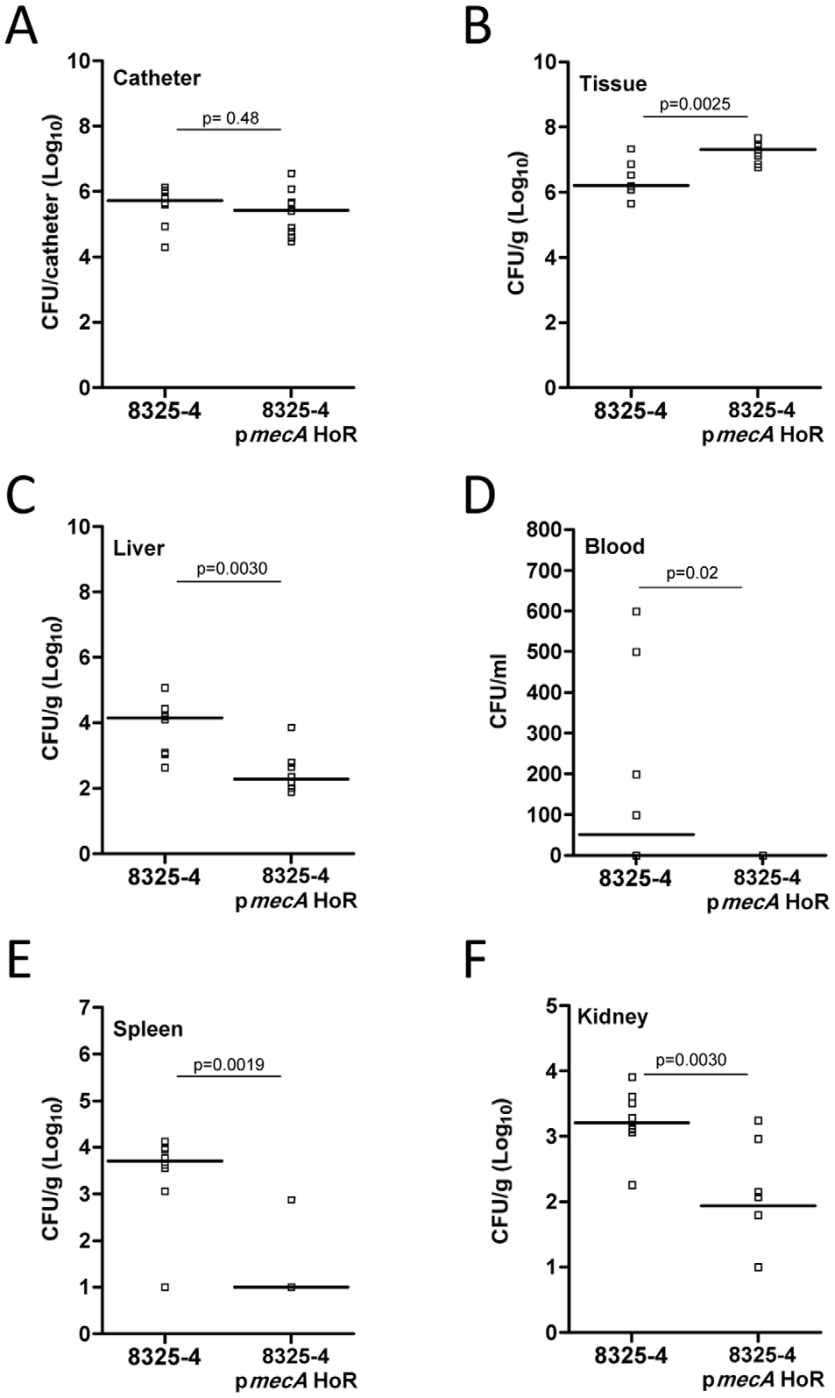
Catheter colonisation and dissemination of 8325-4 and 8325-4 p*mecA* HoR in a mouse device-related infection model. Implanted catheter segments were injected with 1×10^7^ 8325-4 and 8325-4 p*mecA* HoR and animals (*n* = 8 mice per group) were sacrificed after 18 hours. Colony forming units (CFU) per catheter (A), per g peri-catheter tissue (B), per g liver (C), per ml blood (D), per g spleen (E) and per g kidney (F) recovered from animals infected with 8325-4 and 8325-4 p*mecA* HoR. Statistical significance (p values) is indicated.

The high mortality rate associated with infection of 8325-4 made it impossible for us to compare the invasiveness and dissemination of 8325-4 and 8325-4 p*mecA* HoR. Thus, the above experiment was repeated over an 18-hour time period with an inoculum of 1×10^7^ bacteria after which all animals were sacrificed. The implanted catheter sections were aseptically removed and bacteria associated with the implanted biomaterial were quantitatively cultured on tryptone soya agar (TSA). In addition, peri-catheter tissue, liver, kidneys and spleen were dissected, weighed, homogenized and quantitatively cultured on TSA, as were bacteria present in blood. These data revealed that the numbers of adherent bacteria on implanted catheters were similar for 8325-4 and 8325-4 p*mecA* HoR (Figure 7A) indicating that the altered biofilm phenotype expressed by 8325-4 p*mecA* HoR does not diminish its ability to colonize implanted devices. There were significantly more 8325-4 p*mecA* HoR bacteria in the peri-catheter tissue than 8325-4 bacteria (Figure 7B). However significantly fewer 8325-4 p*mecA* HoR bacteria were recovered from the liver, blood, spleen and kidneys than 8325-4 bacteria (Figure 7 C–F). Thus, these findings are consistent with the mortality data and indicate that 8325-4 is significantly more invasive than 8325-4 p*mecA* HoR, which accumulated in the peri-catheter tissue and was less capable of disseminating to other organs.

**Figure 8:**
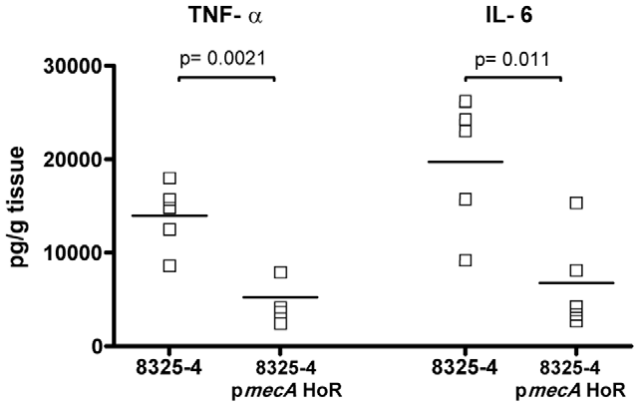
Enzyme-linked immunosorbent assay (ELISA) of TNF-α and IL-6 in peri-catheter tissue recovered from mice (*n* = 7 mice per group) infected for 18 h with 8325-4 and 8325-4 p*mecA* HoR. Statistical significance (p values) is indicated.

The immune response of the mice to 8325-4 and 8325-4 p*mecA* HoR was measured by assaying levels of TNF-α and IL-6 in the peri-catheter tissue. Levels of both pro-inflammatory cytokines were significantly increased in mice inoculated with 8325-4 compared to 8325-4 p*mecA* HoR (Figure 8), despite the fact that there were more 8325-4 p*mecA* HoR cells recovered from the tissue (Figure 7B). Taken together these data reveal that homogeneous, high-level resistance to oxacillin significantly attenuated the virulence of 8325-4.

### Impact of homogeneous methicillin resistance on biofilm production and δ-hemolytic activity in clinical isolates of *S. aureus*

To investigate the impact of homogenous methicillin resistance on biofilm production and Agr activity in clinical *S. aureus* isolates, we first introduced p*mecA* into the MSSA strains MSSA476 and 15981 and isolated HoR derivatives on media containing oxacillin 100 µg/ml. As observed in 8325-4, acquisition of the HoR phenotype in MSSA476 and 15981 was associated with repression of polysaccharide-type biofilm production and expression of a protein adhesin-mediated biofilm phenotype (Figure S4). Furthermore the MSSA476 and 15981 HoR strains exhibited reduced δ-hemolytic activity on sheep blood BHI agar (Figure S4). Given that the δ-hemolysin is encoded by the *hld* gene within the *agr* locus *RNAIII* transcript [41], these data indicate that the HoR phenotype in MSSA476 and 15981 is accompanied by repression of Agr activity.

The impact of the HoR phenotype on biofilm production by the CA-MRSA USA300 strain LAC was also examined. In our experiments this strain formed a protein-adhesin type biofilm in BHI glucose media (Figure S5A). A LAC HoR derivative isolated on media containing oxacillin 100 µg/ml produced significantly more biofilm (Figure S5A) and exhibited reduced δ-hemolytic activity on sheep blood BHI agar (Figure S5B). Finally we examined the impact of the HoR phenotype on three CC5 strains with different SCC*mec* elements exhibiting a heterogeneous pattern of oxacillin resistance, namely DAR173, DAR26 and DAR9. These strains produced a polysaccharide-type biofilm, whereas their HoR derivates isolated on media containing oxacillin 100 µg/ml produced protein adhesin-type biofilms and exhibited reduced δ-hemolytic activity (Figure S5C–K).

In the laboratory strain 8325-4, non-synonymous SNPs in *gdpP* were associated with the HoR phenotype. Similarly, DNA sequencing of the *gdpP* gene from the USA300 LAC HoR derivative identified an R_450_STOP mutation. GdpP amino acid substitutions were also identified in HoR derivatives of the clinical isolates 15981 (P_392_S, D_105_N), MSSA476 (V_52_I, D_105_N, P_392_S) and DAR26 (N_105_D, S_392_P). However these latter substitutions occur in GdpP residues that are not conserved in multiple *S. aureus* species and so their significance is unclear. No *gdpP* mutations were identified in HoR derivatives of the clinical isolates DAR176 and DAR9 suggesting that the HoR phenotypes of these strains may be independent of c-di-AMP or that these strains contain unidentified mutations in c-di-AMP target genes.

## Discussion

Antibiotic resistance, enzyme and toxin production, biofilm forming capacity and immune evasion capability have combined to accelerate the emergence of *S. aureus* as a globally important human pathogen. Our recent research has focused on the relationship between two of these virulence determinants, antibiotic resistance and biofilm-forming capacity, in clinical *S. aureus* isolates. Our data show that MSSA clinical isolates are more likely to produce a PNAG-dependent biofilm than MRSA isolates which produce an Atl/FnBP-dependent biofilm [3], [4], [6], [7], suggesting that methicillin susceptibility influences biofilm expression. MRSA strains contain the *Staphylococcus* cassette chromosome *mec* (SCC*mec*) including the *mecA* gene, which encodes the PBP2a protein that confers resistance to β-lactam antibiotics. To investigate how methicillin resistance influences the biofilm phenotype we introduced a plasmid expressing the *mecA* gene from its own promoter (p*mecA*) into the PNAG-producing laboratory strain 8325-4. 8325-4 p*mecA* exhibited heterogeneous resistance to oxacillin (designated 8325-4 p*mecA* HeR) from which an homogeneous, high-level oxacillin resistant derivative was isolated, designated 8325-4 p*mecA* HoR. Western blots show that expression of homogeneous resistance was associated with substantially increased expression of PBP2a. Genome re-sequencing identified a number of amino acid substitutions and small deletions in the *gdpP* gene of independent 8325-4 HoR strains and in HoR derivatives of the clinical isolates USA300 strain LAC, MSSA476, 15981 and DAR26. GdpP has recently been identified as a phosphodiesterase controlling intracellular levels of the secondary messenger c-di-AMP, which in turn influences phenotypes such as cell wall architecture, biofilm formation and, most relevant to this study, resistance/tolerance to β-lactam antibiotics [37], [38]. Thus c-di-AMP may be involved in the multiple phenotypic changes associated with homogeneous oxacillin resistance. However, given that the GdpP amino acid sequence is identical in 8325-4 (MSSA), USA300 strain LAC (HeR MRSA) and BH1CC (HoR MRSA), mutations in *gdpP* and/or altered c-di-AMP signaling alone may not be sufficient to explain the HeR to HoR transition. Furthermore no *gdpP* SNPs were identified in HoR derivatives of two clinical isolates (DAR9 and DAR176) suggesting that either these strains contain mutations in c-di-AMP target genes or that c-di-AMP-independent mechanisms may also be involved in the HoR phenotype. In this context our recent finding that loss of methicillin resistance in the HoR MRSA strain BH1CC reduced biofilm forming capacity and increased virulence in a mouse sepsis model [42], also indicates that altered c-di-AMP levels alone are unlikely to account for the pleiotropic effects of the HoR phenotype. Clearly homogenous methicillin resistance remains a complex phenotype and future research to identify c-di-AMP targets is necessary to gain mechanistic insights into how this cyclic dinucleotide contributes to the co-regulation of antibiotic resistance, biofilm and virulence.

The biofilm phenotype of 8325-4 p*mecA* HoR was dramatically altered and characterized by the loss of PNAG production and expression of a protein-adhesin mediated biofilm phenotype. Using a series of surface protein mutants, we were able to show that the PBP2a-induced biofilm phenotype was independent of PNAG, Atl, and any of the LPXTG-anchored surface proteins (including the FnBPs and protein A). Furthermore, a monoclonal antibody against PBP2a reduced 8325-4 p*mecA* HoR biofilm production by approximately 50% implicating PBP2a itself in intercellular accumulation. The inability of the PBP2a antibody to fully inhibit 8325-4 p*mecA* HoR biofilm may be explained, at least in part, by our observation that this biofilm phenotype was also dependent on autolytic activity and extracellular DNA. We have previously implicated autolytic activity and eDNA in the early stages of PNAG-independent biofilm phenotypes expressed by *S. aureus* clinical isolates [6], [7]. In contrast biofilm expression by 8325-4 p*mecA* HeR was similar to wild type, indicating that high level PBP2a expression is required to change the biofilm phenotype. In the MRSA strain BH1CC, excision of SCC*mec* and induced methicillin susceptibility also resulted in an impaired biofilm phenotype that could be complemented by the wild type *mecA* gene but not a *mecA* allele expressing a PBP2a mutant with an S_403_A substitution in the active site of the enzyme. Taken together, these data suggest that both high level PBP2a expression and methicillin resistance are required for the PBP2a-induced biofilm phenotype. In addition, because Atl/FnBP-dependent biofilm development by BH1CC was unaffected by PBP2a antibody (data not shown), our data also reveal redundancy among *S. aureus* surface proteins that can promote biofilm development.

**Figure 9:**
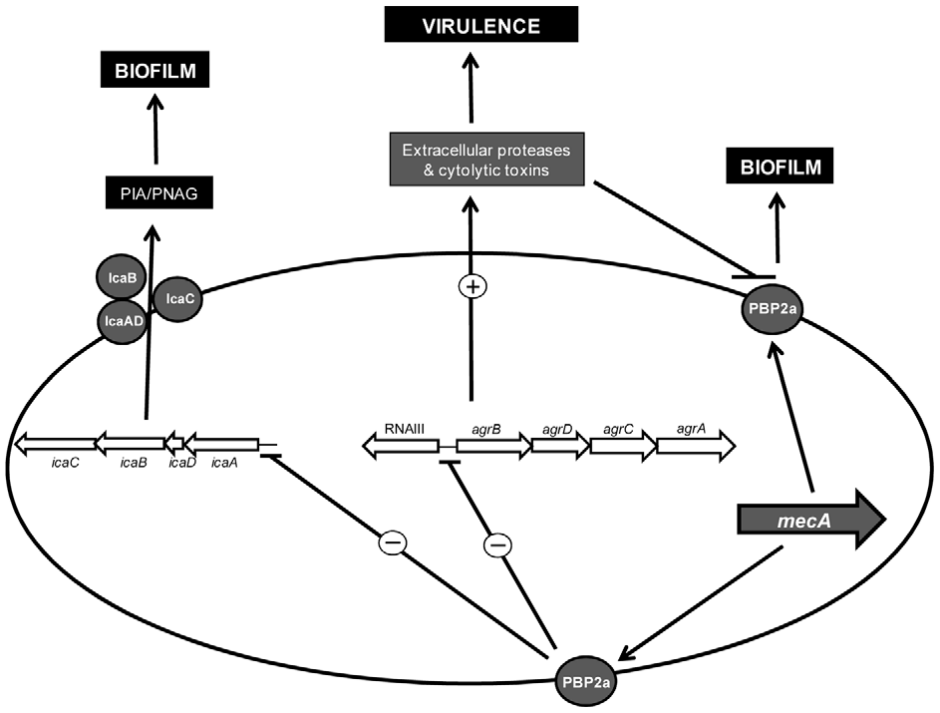
Model of PBP2a-mediated modulation of biofilm expression and virulence in *S. aureus*. High level PBP2a expression and homogeneous methicillin resistance results in repression of the *icaADBC* and *agr* loci blocking PNAG production and reducing expression of extracellular proteases and cytolytic toxins. Reduced levels of extracellular protease and toxin production in turn correlate with PBP2a-promoted biofilm development and attenuated virulence.

How PBP2a promotes biofilm is uncertain but possibly the altered cell wall architecture in methicillin resistant strains expressing high levels of PBP2a may facilitate PBP2a-promoted cell-cell interactions that are not possible between MSSA cells. What is clear, however, is that high level PBP2a expression resulted in significant repression of *icaADBC* transcription and PNAG production in 8325-4 p*mecA* HoR (Figure 9). Comparison of acetic acid levels in 8325-4 and 8325-4 p*mecA* HoR culture supernatants revealed no significant difference (data not shown) suggesting that altered TCA cycle activity, which is known to regulate PIA/PNAG production [43], [44], may not be involved in this phenotype. PBP2a overexpression also resulted in significant repression of the *agr* locus. These data are supported by our recent findings that excision of the SCC*mec* element from the MRSA strain BH1CC and loss of oxacillin resistance had the opposite effect and was associated with increased *agr* transcription [42]. Furthermore our previous data showed that MRSA cells are unable to detect the Agr-encoded auto-inducing peptide (AIP), thus preventing normal activation of the Agr system and concomitant virulence gene regulation [42]. However this does not explain why the *icaADBC* locus is repressed in strains expressing high levels of PBP2a. Given that both the *agr* and *ica* loci are repressed by PBP2a expression, it seems possible that a transcriptional regulator(s) involved in regulating both operons may also play a role in this phenotype. For instance preliminary data indicate that the activity of the virulence and carbon metabolism regulator CcpA, which is known to increase oxacillin resistance levels [45], [46], was activated approx 4-fold in 8325-4 p*mecA* HoR (unpublished findings). On the other hand CcpA positively regulates both *icaADBC* and *agr* expression [45], [46], suggesting that the impact of PBP2a overexpression on biofilm and virulence is complex and experiments are underway to investigate the potential involvement of other transcription factors.

Repression of the Agr locus in response to homogenous methicillin resistance was accompanied by down-regulation of extracellular protease production [35], [47] (Figure 9). Protease activity has previously been shown to alter murein hydrolase activity and in turn biofilm [35], which appears to correlate with our finding implicating autolytic activity in the 8325-4 p*mecA* HoR biofilm phenotype. Thus repression of extracellular proteases combined with high level PBP2a expression and methicillin resistance appears to be required for PBP2a-mediated biofilm development in 8325-4. Consistent with this, activation of *agr* following excision of SCC*mec* from BH1CC increased production of extracellular toxins [42]. Here we have shown that loss of SCC*mec* also increased extracellular protease production in BH1CC, which in turn correlated with diminished Atl/FnBP-promoted biofilm forming capacity. It is also interesting to note that extracellular protease activity was similar in both 8325-4 p*mecA* HoR and BH1CC SCC*mec* p*mecA*, which both express a *mecA*-promoted biofilm phenotype and that as mentioned above BH1CC does not display the characteristic stationary phase induction of RNAIII typical of *agr* ^+^ strains [42]. Taken together these data indicate that the capacity of PBP2a to promote biofilm is dependent, at least in part, on the background levels of extracellular protease activity in individual strains.

The altered biofilm phenotype and repression of *agr* in 8325-4 p*mecA* HoR was accompanied by a significant reduction in virulence in a mouse model of device-related infection. This finding correlated with our previous data that the virulence of BH1CC was increased following excision of SCC*mec* in a mouse sepsis model [42]. Thus using two different strains and two different mouse infection models, our data indicate that expression of methicillin resistance reduces virulence potential in *S. aureus*. Furthermore although the 8325-4 p*mecA* HoR strain was equally capable of colonizing implanted biomaterials, it accumulated significantly more in peri-catheter tissue but was significantly less capable of causing invasive disease, resulting in a significantly reduced mortality rate than the wild type 8325-4. An intriguing backdrop to our findings are the recent reports that *psm-mec* locus, which is located adjacent to the *mecA* locus on type II SCC*mec* elements, also represses virulence and promotes the *S. aureus* biofilm phenotype [26], [27], [28]. Thus both *mecA*-encoded PBP2a and the *psm*-*mec* encoded phenol-soluble modulin-mec (PSM-mec) can independently repress virulence and promote biofilm in MRSA. The *psm-mec*-encoded RNA is able to repress expression of the chromosomally encoded PSMα, while both the *psm-mec* RNA and the PSM-mec protein repress colony spreading ability and promote biofilm [26], [27], [28].

Given that healthcare-associated MRSA strains are typically responsible for infections in immunocompromised patients in which numerous implanted medical devices are used for organ and life support and in which the use of antimicrobial drugs is high, these findings reveal a sophisticated level of adaptation by MRSA to the hospital environment. The reduced metabolic costs associated with PBP2a-induced repression of Agr and exoprotein production coupled with PBP2a-mediated biofilm production, may confer advantages on MRSA strains. Agr defective strains are known to be less virulent [48], [49] and a number of studies [50], [51] have suggested that, in immunocompromised patients, Agr defective strains may have advantages including reduced metabolic costs of exoprotein production, increased biofilm production [52] and increased FnBP expression [53] with resulting effects on host cell invasion/immune evasion [54]. Indeed biofilm-forming *S. epidermidis* strains, which express fewer exoproteins and exotoxins (and are consequently less virulent), are also significant pathogens in this patient group. Thus sacrificing virulence for antibiotic resistance is not necessarily a disadvantage for MRSA and may in fact benefit the pathogen in this clinical setting. Furthermore MRSA strains have retained the capacity for biofilm production, albeit using surface protein adhesins rather than PNAG, which is important given that the majority of bloodstream infections in hospital patients are associated with implanted medical devices.

## Materials and Methods

### Ethics statement

This study was carried out in strict accordance with the recommendations in the Guide for the Care and Use of Laboratory Animals of the National Institutes of Health. All mouse protocols were reviewed and approved by the Institutional Animal Care and Use Committee at the University of Nebraska Medical Centre. All surgery was performed under anesthesia and all efforts were made to minimize suffering.

### Bacterial strains, plasmids and growth conditions

The *S. aureus* strains and the plasmids used in the manipulation of these strains are described in Table 1. *Escherichia coli* strains were grown at 37°C on LB medium supplemented, when required, with ampicillin (100 µg/ml) or kanamycin (50 µg/ml). *S. aureus* strains were grown at 30°C or 37°C on Brain-Heart Infusion (BHI) (Oxoid) medium supplemented when required with chloramphenicol (10 µg/ml), tetracycline (10 µg/ml), kanamycin (10 µg/ml) and oxacillin (0–100 µg/ml). BHI broth was supplemented where indicated with 1% glucose or 4% NaCl.

**Table 1:** Strains and plasmids used in this study.

Strain/Plasmid	Characteristic(s)	Source/reference
8325-4	NCTC8325 cured of prophages. 11-bp deletion in *rsbU.*	[33]
RN4220	Restriction deficient mutant of 8325-4.	[64]
SH1000	8325-4 with repaired defect in *rsbU.*	[33]
HG003	NCTC8325 with repaired *tcaR* and *rsbU* genes.	[36]
8325-4 p*mecA* HeR	8325-4 carrying p*mecA* expressing heterogeneous resistance to oxacillin.	[42]
8325-4 p*mecA* HoR	8325-4 carrying p*mecA* expressing homogeneous resistance to oxacillin.	This study
ICA1	Δ*icaADBC*::Tc^r^; isogenic mutant of 8325-4.	[3]
DU5883	*fnbA*::Tc^r^,*fnbB*::Em^r^; isogenic mutant of 8325–4.	[65]
SKM1	Δ*srtA::*Em^r^; isogenic mutant of RN4220.	[66]
JT1392	Δ*atl::*Cm^r^, isogenic mutant of RN4220.	[67]
*spa*::Km^r^	*spa*::Km^r^, isogenic mutant of 8325-4.	[68]
BH1CC	MRSA clinical isolate. Biofilm positive. SCC*mec* type II, MLST type 8, clonal complex 8.	[4]
BH1CC ΔSCC*mec*	Isogenic mutant of BH1CC lacking SCC*mec*.	[61]
BH10(03)	Biofilm positive, homogenoeously methicillin resistant MRSA. SCC*mec* type IV, clonal complex 22.	[4]
DAR13	Biofilm positive, homogenoeously methicillin resistant (HoR) MRSA. SCC*mec* type IV, clonal complex 8.	[4]
DAR168	Biofilm positive, HoR MRSA. SCC*mec* type I, clonal complex 8.	[4]
DAR34	Biofilm positive, HoR MRSA. SCC*mec* type II, clonal complex 8.	[4]
DAR199	Biofilm positive, HoR MRSA. SCC*mec* type II, clonal complex 30.	[4]
DAR112	Biofilm positive, HoR MRSA. SCC*mec* type III, clonal complex 239.	[4]
DAR9	Biofilm positive, heterogeneously methicillin resistant (HeR) MRSA. SCC*mec* type I, clonal complex 5.	[4]
DAR26	Biofilm positive, HeR MRSA. SCC*mec* type IV, clonal complex 5.	[4]
DAR173	Biofilm positive, HeR MRSA. SCC*mec* type II, clonal complex 5.	[4]
MSSA476	Community associated-MSSA. Novel SCC element lacking *mecA*.	[69]
LAC	Community associated, HeR MRSA USA300.	[35]
15981	Clinical MSSA isolate. Biofilm positive. *rsbU* ^+.^	[70]
*E. coli* TOPO	*recA1 endA1 lac* [F′ *proAB lac1q* Tn10 (tet^r^)]	Invitrogen
**Plasmids**		
pSR	Shuttle vector carrying the SCC*mec ccrA* and *ccrB* recombinase genes	[15]
pRB474	Low copy *E. coli-Staphylococcus* shuttle vector. Ap^r^ (*E. coli*), Cm^r^ (*Staphylococcus*).	[71]
pSA*mecA*5	2,850 bp PCR fragment containing *mecA* gene amplified using primers SAmecA3 and SAmecA4 and cloned into pCR-Blunt II-TOPO (Invitrogen).	[42]
p*mecA*	2,867 bp *Eco*RI fragment containing *mecA* from pSA*mecA*5 subcloned into the *Eco*RI site of pRB474.	[42]
p*mecA*S403A	p*mecA* expressing a PBP2a S403A mutant	This study

δ-haemolytic activity was visualized on sheep blood BHI agar as described by Traber et al. [41]. Briefly clinical *S. aureus* isolates were inoculated onto a lawn of *S. aureus* RN4220, which produces only β-haemolysin. The RN4220-expressed β-haemolysin enhances lysis of red blood cells by δ-haemolysin, while inhibiting α-haemolysin, thus enabling detection of δ-haemolytic activity by clinical *S. aureus* isolates.

### Genetic techniques

Genomic and plasmid DNA was prepared using Wizard Genomic DNA and plasmid purification kits (Promega). Prior to DNA extraction cells were pre-treated with 5–10 µl of a 1 mg/ml concentration of lysostaphin (Ambi products, New York) in 100 µl 50 mM EDTA to facilitate lysis. Restriction and DNA modifying enzymes (Roche, UK and New England Biolabs, MA) were used according to the manufacturer's instructions. The serine residue at amino acid 403 in the active site of PBP2a was mutated to alanine using Phusion polymerase (NEB) and the primers mecAS403A_For and mecAS403A_Rev (Table 2). Plasmid p*mecA* was used as the template. Successful mutagenesis of the DNA sequence encoding the S403 residue resulted in the introduction of a new *Dra*III restriction enzyme site and candidate plasmids harbouring the mutation were digested with this enzyme before being confirmed by sequencing. The mutated plasmid designated p*mecA*S403A.

**Table 2:** Oligonucleotide primers used in this study.

Target gene	Primer names	Primers sequence (5′-3′)
*mecA*	SAmecA1	AGTTGTAGTTGTCGGGTTTGG
	SAmecA2	GCATTGTAGCTAGCCATTCCTT
*mecA*	SAmecA3	TGACGATTCCAATGACGAAC
	SAmecA4	GCATCTGGCATGCATACACT
*mecA*	SAmecAFOR	TTGGAACGATGCCTATCTCA
	SAmecAREV	TCCAGGAATGCAGAAAGACC
16S rRNA	SA_16S_for	ATGCAAGTCGAGCGAAC
	SA_16S_rev	TGTCTCAGTTCCAGTGTGGC
RNAIII	RNAIII_2_for	GGAGTGATTTCAATGGCACA
	RNAIII_2_rev	CATGGTTATTAAGTTGGGATGG
*icaA*	SAicaEON1	TACCTTGCCTAACCCGTAC
	SAicaEON2	GTTGGCTCAATGGGGTCTAA
*icaR*	SAicaR1	TTCTCAATATCGATTTGTATTGTCAAC
	SAicaR2	TGTCAGGCTTCTTGTTCAATG
*mecA*	mecAS403_For	ACAACTTCACCAGGT**TGC**ACTCAAAAAATATTA
	mecAS403R_Rev	TAATATTTTTTGAGT**GCA**ACCTGGTGAAGTTGT
*gdpP*	gdpP_For	CGCATGGTTCAGACGATAAA
	gdpP_Rev	GCTTCGGCAATTTGTTTTGT

Transformations of plasmid DNA into *E. coli* and *Staphylococcus* strains were performed as described previously [55]. MWG Biotech, Germany or Sigma-Aldrich, Ireland supplied oligonucleotide primers used for PCR and RT-PCR (Table 2).

### Biofilm and protease assays

Semi-quantitative measurements of biofilm formation were determined using Nunclon tissue culture treated (Δ surface) 96-well polystyrene plates (Nunc, Denmark), based on the methods of Christensen et al. [56] and Ziebuhr et al. [57] with the following modification. Bacteria were grown in individual wells of 96-well plates at 37°C in BHI medium or BHI supplemented with 4% NaCl or 1% glucose. The serine protease inhibitor dichloroisocoumarin was added to BHI glucose media at concentrations of 0.004–0.5 mM, where indicated. After 24 h of growth, the plates were washed vigorously three times with distilled H_2_O to remove unattached bacteria and dried for 1 hour at 60°C, as recommended by Gelosia et al. [58] as described previously [4], [55]. The absorbance of the adhered, stained biofilms was measured at *A*_492_ using a microtitre plate reader. Each strain was tested at least three times and average results are presented. Mouse monoclonal anti-PBP2a antibody (CalBioreagents, CA), DNase I (Sigma) or polyanethole sodium sulfonate (PAS) (Sigma) were added to biofilm cultures at the start of the assay as indicated. Biofilm stability against proteinase K (Sigma), sodium-meta-periodate (Sigma) was tested as described previously [10], [59], [60].

Bacteria were grown on Congo red agar (CRA) plates, which are composed of BHI agar supplemented with 5% sucrose (Sigma) and 0.8 mg of Congo red/ml (Sigma) to distinguish between PNAG-producing (black, dry colony morphology) and non-PNAG-producing (red, smooth colony morphology) phenotypes as described previously [4], [55].

Protease activity in culture supernatants was measured using a protease assay kit (Calbiochem, Germany) according to the manufacturer's instructions.

To analyze biofilm formation under flow conditions, we utilized the BioFlux 1000 microfluidic system (Fluxion Biosciences Inc., South San Francisco, CA) which allows automated image acquisition within specialized multi-well plates. To grow biofilms, the microfluidic channels were primed with 50% BHI supplemented with 4% NaCl or 1% glucose at 10.0 dyn/cm^2^. Channels were seeded at 2 dyn/cm^2^ with 10^7^ CFU from overnight cultures of 8325-4 p*mecA* HeR and 8325-4 p*mecA* HoR. The plate was then incubated at 37°C for 1 hour to allow cells to adhere. Excess inoculums were removed and 2 ml of 50% BHI supplemented with 4% NaCl or 1% glucose was added to the input wells. Biofilms were grown at 37°C with a flow of fresh media at a constant shear of 0.7 dyn/cm^2^. Images were taken every 5 minutes for 18 hours at 200× magnification under brightfield.

### Isolation and analysis of a homogeneous oxacillin resistant derivative of *S. aureus* 8325-4

The p*mecA* plasmid [61] was introduced by electroporation into 8325-4. The 8325-4 p*mecA* strain exhibited heterogeneous oxacillin resistance (HeR) characterized by a minority of cells with an MIC>100 µg/ml and the majority of cells with an MIC<1 µg/ml. To obtain high-level oxacillin resistant derivatives, dilutions of 8325-4 p*mecA* cultures were grown on BHI agar containing increasing concentrations of oxacillin (0–100 µg/ml) and the number of colony forming units counted after 24 h growth at 37°C. High-level, homogeneous oxacillin resistant derivatives (HoR) were subcultured from oxacillin 100 µg/ml plates. Curing the p*mecA* plasmid from 8325-4 p*mecA* HoR was achieved by 48 h growth in antibiotic free media at 45°C and isolation of chloramphenicol susceptible colonies followed by plasmid profile analysis.

The genomes of the 8325-4 p*mecA* HeR and 8325-4 p*mecA* HoR were sequenced using an Illumina Genome Analyzer as per manufacturer's instructions (Illumina, San Diego, CA, USA) generating 5.7 million and 7.5 million reads for 8325-4 p*mecA* HoR and 8325-4 p*mecA* HeR, respectively. The genome sequences were then mapped back to the *S. aureus* NCTC8325 (CP000253) genome sequence using the short read aligner Bowtie (http://bowtie-bio.sourceforge.net/index.shtml & PMID:19261174) allowing up to 2 mismatches per uniquely mapped read. Single nucleotide polymorphisms (SNPs) were identified using samtools programs [PMID:19505943] in regions with a read depth of greater than 4. Identified SNPs were confirmed by PCR amplification followed by capillary electrophoresis sequencing of all candidate polymorphic regions from 8325-4 p*mecA* HeR and 8325-4 p*mecA* HoR.

### RNA purification and real time RT-PCR

Cultures were grown in BHI glucose after which cells were collected and immediately stored at −20°C in RNA*later* (Ambion) to ensure maintenance of RNA integrity prior to purification. Cells were pelleted and resuspended in 100 µl of 200 mM Tris HCl (pH 7.8) supplemented with 800 µg/ml lysostaphin for 2 min to weaken the cell wall prior to lysis. Total RNA was subsequently isolated using the Qiagen RNeasy mini kit (Qiagen) according to the manufacturer's instructions. Residual DNA present in the RNA preparations was removed using Ambion recombinant Turbo DNase. Purified RNA was eluted and stored in RNA*secure* resuspension solution (Ambion) and the integrity of the rRNA confirmed by agarose gel electrophoresis. RNA concentration was determined using a Nanodrop spectrophotometer.

Real-time reverse transcription-PCR (RT-PCR) was performed on a LightCycler instrument using the RNA amplification kit Sybr Green I (Roche Biochemicals, Switzerland) following the manufacturer's recommended protocol. RT was performed at 61°C for 30 min, followed by a denaturation step at 95°C for 30 sec and 35 amplification cycles of 95°C for 20 sec, 50°C for 20 sec and 72°C for 20 sec. Melting curve analysis was performed at 45°C to 95°C (temperature transition, 0.1°C per sec) with stepwise fluorescence detection. For LightCycler RT-PCR, RelQuant software (Roche Biochemicals) was used to measure relative expression of target genes. 16S rRNA was used as an internal standard in real-time RT-PCR experiments. Each experiment was performed at least three times and average data with standard deviations are presented.

### PNAG assays

PNAG assays were performed as described elsewhere [62]. Briefly 5 ml overnight cultures (approximately 5×10^9^ bacteria) were collected by centrifugation, resuspended in 500 µl of 0.5 M EDTA and boiled for 5 min. The cell debris was again centrifuged and the supernatant treated with 20 µg proteinase K at 65°C for 1 h. The proteinase K was inactivated by boiling for 5 min and the samples diluted as appropriate before application onto nitrocellulose (pre-wetted in TBS) using a vacuum blotter. The blots were dried, re-wet in TBS, and blocked for 1 h in 5% skimmed milk. The primary antibody (1∶5,000 dilution of rabbit anti-PNAG (a kind gift from Tomas Maira Litran) in TBST+1% skimmed milk) was then applied to the membrane for 1 h. Horseradish peroxidase linked anti-rabbit IgG secondary antibody (1∶5,000 dilution in TBST+1% skimmed milk) was then incubated with the membrane for 1 h. A chemiluminescence kit (Amersham) was used to generate light via the HRP-catalyzed breakdown of luminal and detected using a BioRad Fluor-S Max CCD camera system.

### Detection of PBP2a by Western blot

Western blot analysis of PBP2a was performed as described elsewhere [63] using overnight bacterial cultures (20 ml) grown at 37°C in BHI glucose. The *A*_600_ of the overnight cultures was measured to ensure that similar cell densities were present prior to protein extraction. The cells were harvested by centrifugation, washed with 50 mM Tris (Sigma), 150 mM NaCl and 5 mM MgCl (pH 7.5) and resuspended in the same buffer. Lysostaphin (200 µg/µl), RNase (10 µg/µl) and DNase (20 µg/µl) were added to the cell suspension and incubated at 37°C for 30 min. The cells were disrupted by sonication on ice and the insoluble cell fraction was pelleted by ultracentrifugation at 80000× *g* for 40 min before being resuspended in 50 mM sodium phosphate (pH 7.0) containing 6 M urea.

The membrane proteins (25 µg) were separated using 10% sodium dodecyl sulfate-polyacrylamide gel electrophoresis and electrophoretically transferred onto Immobilon-P membranes (Millipore) at 12 V for 30 mins. The primary antibody (1∶5000 dilution of a mouse anti-PBP2a monoclonal antibody (Denka Seiken) in TBS+0.1% Tween) was applied to the membranes overnight. A horseradish peroxidase conjugated anti-mouse IgG secondary antibody (1∶1000 in TBST+1% skim milk) was incubated with the membranes for 1 hour. A chemiluminescence kit (Amersham) was used as described above.

### Mouse infection experiments

A mouse model of device-related infection [39] was used to compare virulence of *S. aureus* 8325-4 and 8325-4 p*mecA* HoR. Briefly, the flanks of anesthetized 6-week old male C57BL/6 mice were shaved, and the skin cleansed with povidone-iodine. Using aseptic technique, a 1-cm segment of 14-gauge polyethylene intravenous catheter was implanted into the subcutaneous space (two per mouse and the incision closed with Vetbond (3M, Minneapolis, MN). Next, 1×10^7^ or 1×10^8^ *S. aureus* were injected into the catheter lumen. Eight mice were used to test each inoculum and strain. Survival over 7 days was measured. Animals were euthanized before the end of the experiment using the following indices for evaluating whether a moribund state had been achieved: extreme lethargy, failure to demonstrate typical avoidance behaviour when handled, ulceration of the infection site through the skin, excessive loss of body weight (i.e. >20%), and/or labored breathing.

The high level of mortality associated with infection of 8325-4 prompted us to repeat the above experiment over 18 hours using an inoculum of 1×10^7^ bacteria before sacrificing all animals and enumerating the numbers of bacteria associated with the catheter, surrounding tissue, blood, liver, spleen and kidneys. To do this, the catheters were aseptically removed, placed in sterile microcentrifuge tubes with 1 ml of phosphate buffered saline (PBS), vortexed for one minute, and quantitatively cultured on tryptone soya agar (TSA). In addition, peri-catheter tissue, liver, kidneys and spleen were dissected, weighed, homogenized and quantitatively cultured on TSA. Finally, bacteria present in blood were also quantitatively cultured on TSA.

TNF-α and IL-6 levels were measured in the peri-catheter tissue using TNF-α (OptiEIA, BD Bioscience) or IL-6 (Duoset; R&D Systems) ELISA kits according to the manufacturer's instructions. Results were normalized to the total amount of tissue recovered.

### Statistical analysis

Two-tailed, two-sample equal variance Student's t-tests (Microsoft Excel 2007) were used to determine statistically significant differences in biofilm forming capacity and relative gene expression.

For the animal experiments descriptive statistics (including mean, standard deviation, median minimum and maximum) values were calculated for each strain at each location i.e. catheter, peri-catheter tissue, blood, liver, spleen and kidneys. Log10 transformation of CFU data from catheter and tissue was used to ensure normal distribution. A two-sample t-test was conducted to compare the log-transformed CFU values for 8325-4 and 8325-4 p*mecA* HoR from either catheter or peri-catheter tissue. Nonparametric Wilcoxon rank sum tests were used to compare 8325-4 and 8325-4 p*mecA* HoR CFU data from blood, liver, spleen and kidneys.

## Supporting Information

Figure S1 Biofilm phenotypes of DAR13 (CC8, SCC*mec* type IV), DAR168 (CC8, SCC*mec* type I), DAR34 (CC8, SCC*mec* type II), BH10(03) (CC22, SCC*mec* type IV), DAR199 (CC30, SCC*mec* type II) and DAR112 (CC239, SCC*mec* type III), together with their respective ΔSCC*mec* mutants and the ΔSCC*mec* mutants complemented with p*mecA*. Biofilms were grown for 24 h at 37°C in BHI glucose in hydrophilic 96-well polystyrene plates. The data presented are the average of three independent experiments and standard deviations are shown.
(TIF)

Figure S2 Immunoblot analysis of PNAG production in whole cell extracts of 8325-4, HG003 and 8325-4 Δ*icaADBC* grown overnight at 37°C in BHI media.
(TIF)

Figure S3 Biofilm formation by 8325-4 p*mecA* HeR and 8325-4 p*mecA* HoR under flow conditions after 15 h growth in BHI glucose or BHI NaCl. Biofilms were grown in a BioFlux 1000 microfluidics system. Images were taken at 200× magnification under brightfield illumination.
(TIF)

Figure S4 Impact of homogeneous oxacillin resistance on the biofilm phenotypes and δ-hemolytic activity of the MSSA strains 15981 and MSSA476. (A) Biofilm phenotypes of 15981, 15981 p*mecA* HeR, 15981 p*mecA* HoR and 15981 p*mecA* HoR (cured). (B) δ hemolytic activity of 15981 p*mecA* HeR and 15981 p*mecA* HoR on sheep blood agar (C) Dispersal of 5981, 15981 p*mecA* HeR, 15981 p*mecA* HoR and 15981 p*mecA* HoR (cured) biofilms by sodium metaperiodate and proteinase K. (D) Biofilm phenotypes of MSSA476, MSSA476 p*mecA* HeR, MSSA476 p*mecA* HoR and MSSA476 p*mecA* HoR (cured). (E) δ hemolytic activity of MSSA476 p*mecA* HeR and MSSA476 p*mecA* HoR on sheep blood agar. (F) Dispersal of MSSA476, MSSA476 p*mecA* HeR, MSSA476 p*mecA* HoR and MSSA476 p*mecA* HoR (cured) biofilms by sodium metaperiodate and proteinase K. All biofilms were grown for 24 h in BHI, BHI NaCl and BHI glucose on hydrophilic polystyrene. Experiments were repeated three times and average data are shown.
(TIF)

Figure S5 Impact of homogeneous oxacillin resistance on the biofilm phenotypes and δ-hemolytic activity of HeR MRSA strains. (A) Dispersal of USA300 strain LAC and USA300 strain LAC HoR biofilms by sodium metaperiodate and proteinase K. (B) δ hemolytic activity of USA300 HeR and USA300 HoR on sheep blood agar. (C and D) Dispersal of DAR26 wild type (HeR, CC5, SCC*mec* type IV) and DAR26 HoR biofilms by sodium metaperiodate and proteinase K. (E) δ hemolytic activity of DAR26 HeR and DAR26 HoR on sheep blood agar. (F and G) Dispersal of DAR173 wild type (HeR, CC5, SCC*mec* type II) and DAR173 HoR biofilms by sodium metaperiodate and proteinase K. (H) δ hemolytic activity of DAR173 HeR and DAR173 HoR on sheep blood agar. (I and J) Dispersal of DAR9 wild type (HeR, CC5, SCC*mec* type and DAR9 HoR biofilms by sodium metaperiodate and proteinase K. (K) δ hemolytic activity of DAR9 HeR and DAR9 HoR on sheep blood agar. HoR strains were isolated and cultured in oxacillin 100 µg/ml-supplemented media. USA300 strain LAC biofilms were grown in BHI glucose only. DAR26, DAR173 and DAR9 biofilms were grown in BHI, BHI NaCl and BHI glucose. All biofilms were grown for 24 h at 37°C on hydrophilic polystyrene. Experiments were repeated three times and average data are shown.
(TIF)
