# Supplementary figures and images for: Methicillin Resistance Alters the Biofilm Phenotype and Attenuates Virulence in Staphylococcus aureus Device-Associated Infections
Source: PLoS Pathog. 2012 Apr 5;8(4):e1002626. doi: 10.1371/journal.ppat.1002626 (PMC3320603; doi:10.1371/journal.ppat.1002626)

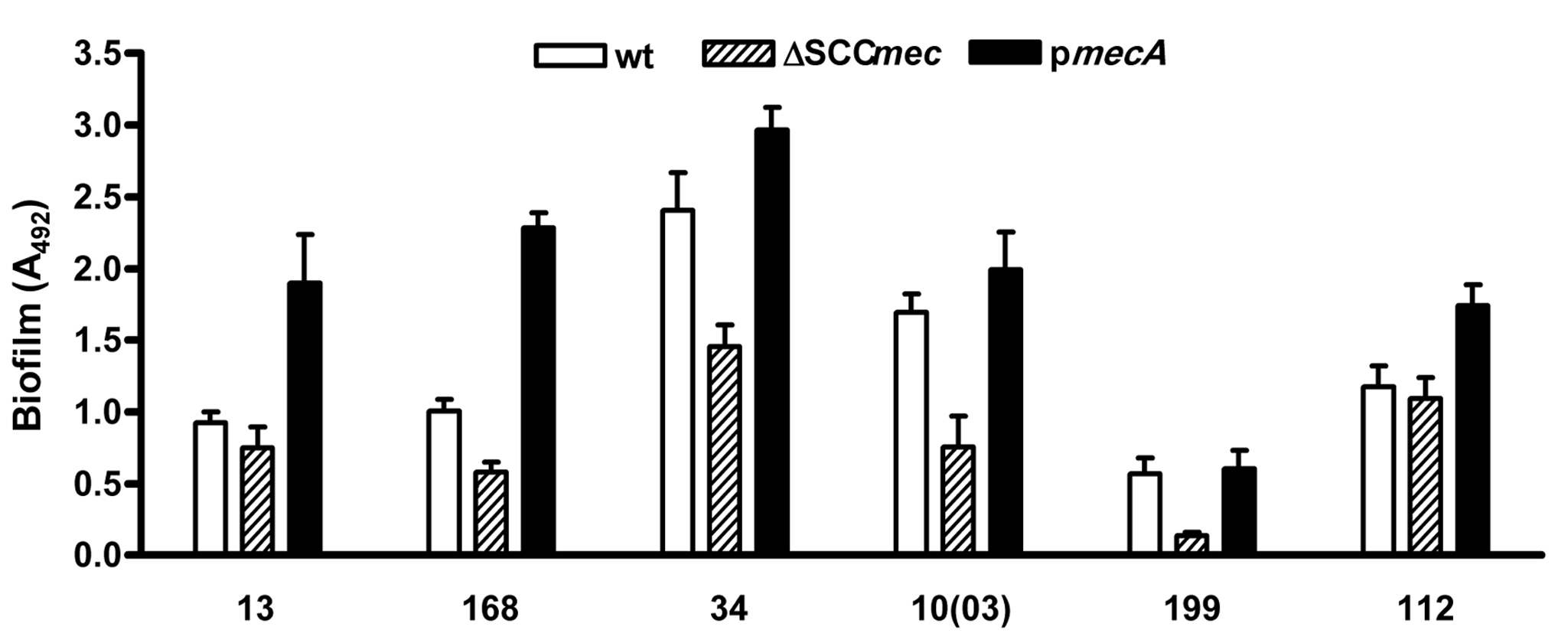

Supplement: Figure S1 — Biofilm phenotypes of DAR13 (CC8, SCCmec type IV), DAR168 (CC8, SCCmec type I), DAR34 (CC8, SCCmec type II), BH10(03) (CC22, SCCmec type IV), DAR199 (CC30, SCCmec type II) and DAR112 (CC239, SCCmec type III), together with their respective ΔSCCmec mutants and the ΔSCCmec mutants complemented with pmecA. Biofilms were grown for 24 h at 37°C in BHI glucose in hydrophilic 96-well polystyrene plates. The data presented are the average of three independent experiments and standard deviations are shown. (TIF) [file ppat.1002626.s001.tif]

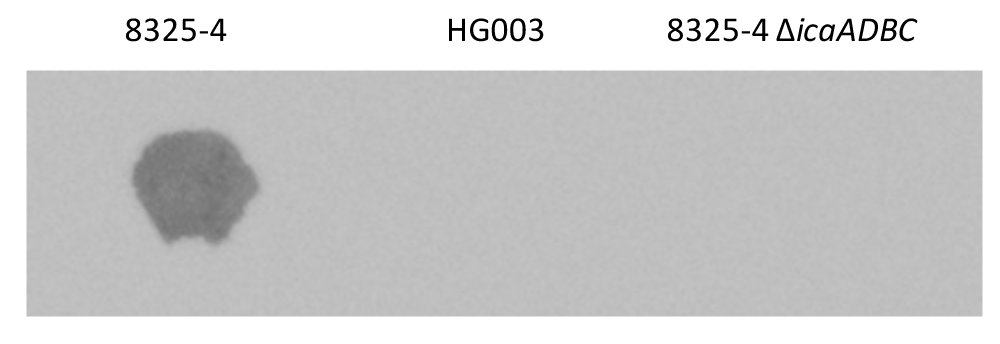

Supplement: Figure S2 — Immunoblot analysis of PNAG production in whole cell extracts of 8325-4, HG003 and 8325-4 ΔicaADBC grown overnight at 37°C in BHI media. (TIF) [file ppat.1002626.s002.tif]

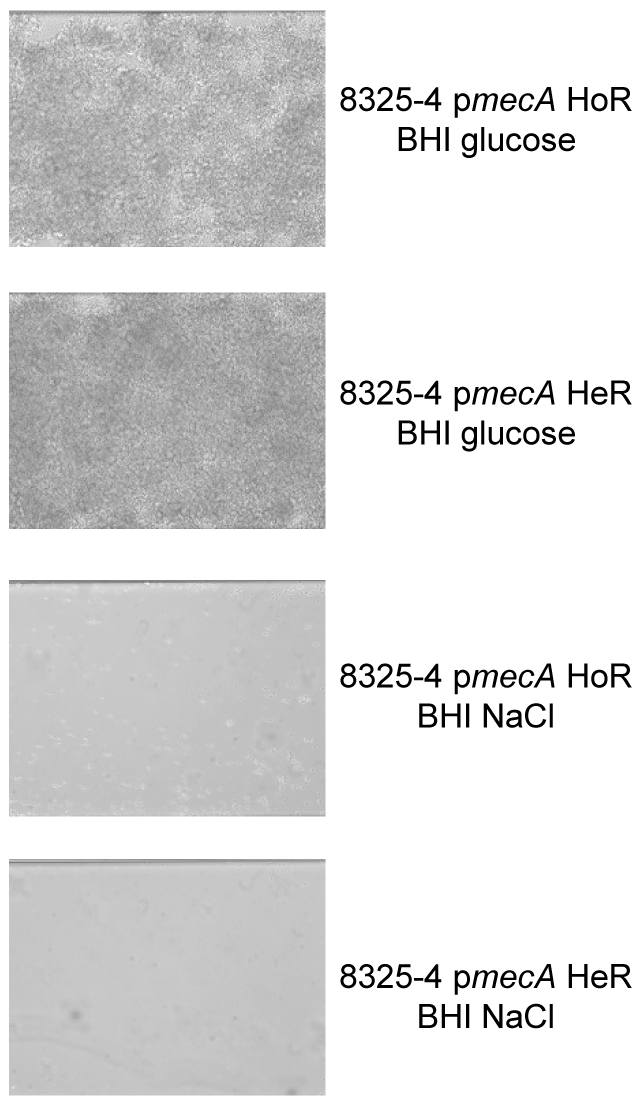

Supplement: Figure S3 — Biofilm formation by 8325-4 pmecA HeR and 8325-4 pmecA HoR under flow conditions after 15 h growth in BHI glucose or BHI NaCl. Biofilms were grown in a BioFlux 1000 microfluidics system. Images were taken at 200× magnification under brightfield illumination. (TIF) [file ppat.1002626.s003.tif]

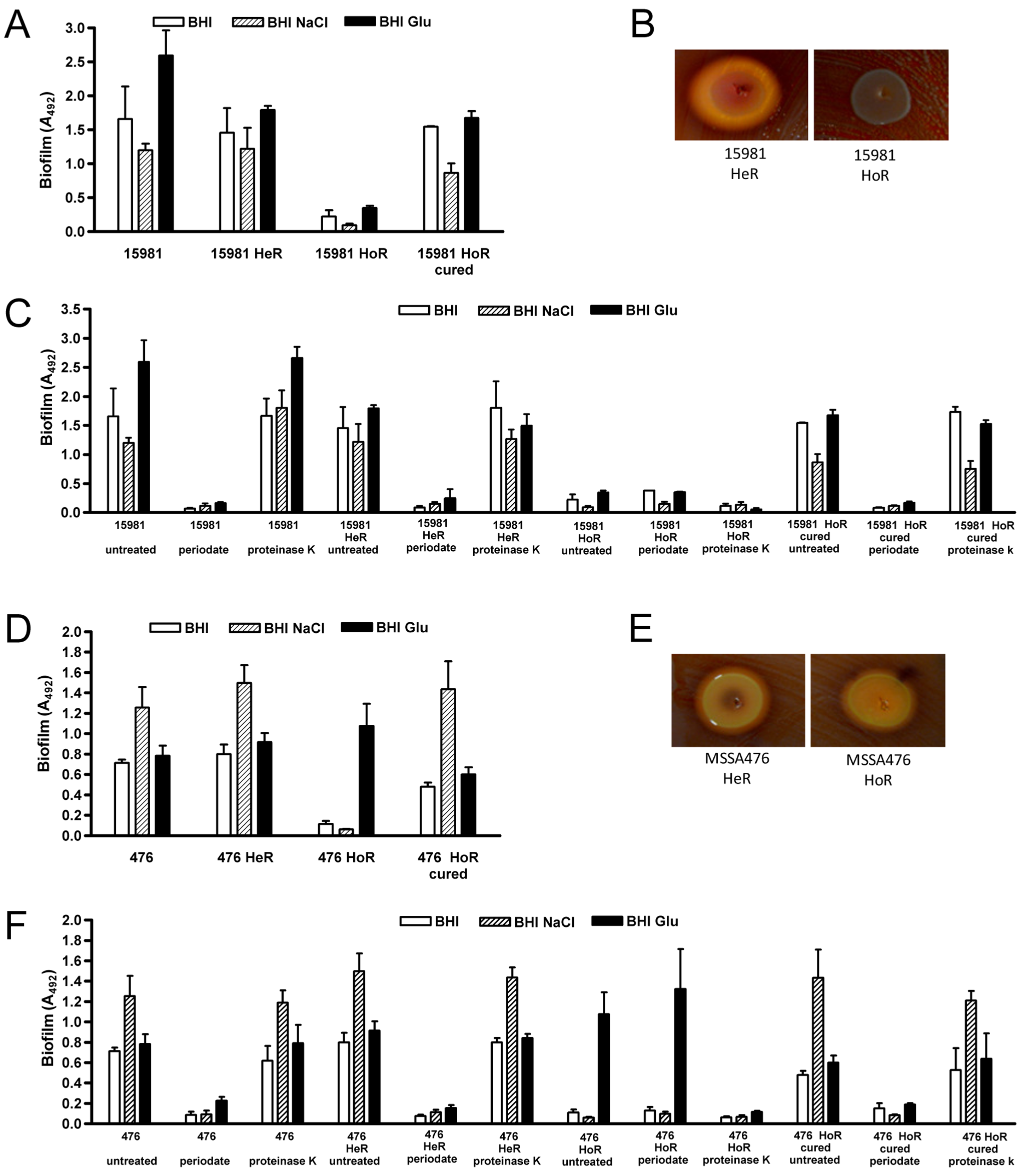

Supplement: Figure S4 — Impact of homogeneous oxacillin resistance on the biofilm phenotypes and δ-hemolytic activity of the MSSA strains 15981 and MSSA476. (A) Biofilm phenotypes of 15981, 15981 pmecA HeR, 15981 pmecA HoR and 15981 pmecA HoR (cured). (B) δ hemolytic activity of 15981 pmecA HeR and 15981 pmecA HoR on sheep blood agar (C) Dispersal of 5981, 15981 pmecA HeR, 15981 pmecA HoR and 15981 pmecA HoR (cured) biofilms by sodium metaperiodate and proteinase K. (D) Biofilm phenotypes of MSSA476, MSSA476 pmecA HeR, MSSA476 pmecA HoR and MSSA476 pmecA HoR (cured). (E) δ hemolytic activity of MSSA476 pmecA HeR and MSSA476 pmecA HoR on sheep blood agar. (F) Dispersal of MSSA476, MSSA476 pmecA HeR, MSSA476 pmecA HoR and MSSA476 pmecA HoR (cured) biofilms by sodium metaperiodate and proteinase K. All biofilms were grown for 24 h in BHI, BHI NaCl and BHI glucose on hydrophilic polystyrene. Experiments were repeated three times and average data are shown. (TIF) [file ppat.1002626.s004.tif]

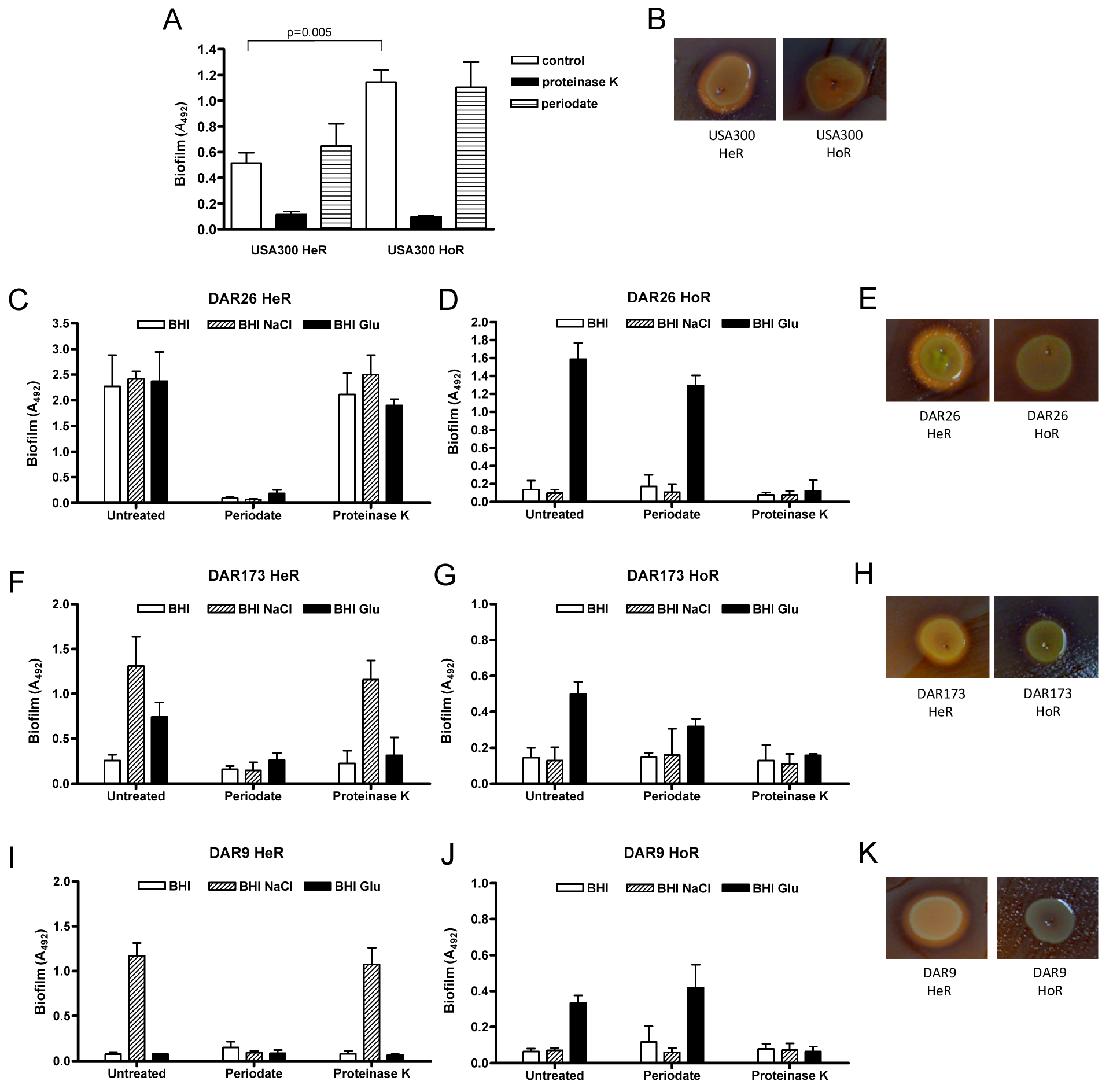

Supplement: Figure S5 — Impact of homogeneous oxacillin resistance on the biofilm phenotypes and δ-hemolytic activity of HeR MRSA strains. (A) Dispersal of USA300 strain LAC and USA300 strain LAC HoR biofilms by sodium metaperiodate and proteinase K. (B) δ hemolytic activity of USA300 HeR and USA300 HoR on sheep blood agar. (C and D) Dispersal of DAR26 wild type (HeR, CC5, SCCmec type IV) and DAR26 HoR biofilms by sodium metaperiodate and proteinase K. (E) δ hemolytic activity of DAR26 HeR and DAR26 HoR on sheep blood agar. (F and G) Dispersal of DAR173 wild type (HeR, CC5, SCCmec type II) and DAR173 HoR biofilms by sodium metaperiodate and proteinase K. (H) δ hemolytic activity of DAR173 HeR and DAR173 HoR on sheep blood agar. (I and J) Dispersal of DAR9 wild type (HeR, CC5, SCCmec type and DAR9 HoR biofilms by sodium metaperiodate and proteinase K. (K) δ hemolytic activity of DAR9 HeR and DAR9 HoR on sheep blood agar. HoR strains were isolated and cultured in oxacillin 100 µg/ml-supplemented media. USA300 strain LAC biofilms were grown in BHI glucose only. DAR26, DAR173 and DAR9 biofilms were grown in BHI, BHI NaCl and BHI glucose. All biofilms were grown for 24 h at 37°C on hydrophilic polystyrene. Experiments were repeated three times and average data are shown. (TIF) [file ppat.1002626.s005.tif]
